# The SUN-like protein TgSLP1 is essential for nuclear division in the apicomplexan parasite *Toxoplasma gondii*

**DOI:** 10.1242/jcs.260337

**Published:** 2023-10-30

**Authors:** Mirjam Wagner, Yuan Song, Elena Jiménez-Ruiz, Sonja Härtle, Markus Meissner

**Affiliations:** ^1^ Experimental Parasitology, Department of Veterinary Sciences, Faculty of Veterinary Medicine, Ludwig-Maximilians-Universität, LMU, Munich, 82152, Planegg, Germany; ^2^ Department of Veterinary Sciences, Faculty of Veterinary Medicine, Ludwig-Maximilians-Universität, LMU, Munich, 82152, Planegg, Germany

**Keywords:** Replication, SUN protein, LINC, Parasite, *Toxoplasma*

## Abstract

Connections between the nucleus and the cytoskeleton are important for positioning and division of the nucleus. In most eukaryotes, the linker of nucleoskeleton and cytoskeleton (LINC) complex spans the outer and inner nuclear membranes and connects the nucleus to the cytoskeleton. In opisthokonts, it is composed of Klarsicht, ANC-1 and Syne homology (KASH) domain proteins and Sad1 and UNC-84 (SUN) domain proteins. Given that the nucleus is positioned at the posterior pole of *Toxoplasma gondii*, we speculated that apicomplexan parasites must have a similar mechanism that integrates the nucleus and the cytoskeleton. Here, we identified three UNC family proteins in the genome of the apicomplexan parasite *T. gondii*. Whereas the UNC-50 protein TgUNC1 localised to the Golgi and appeared to be not essential for the parasite, the SUN domain protein TgSLP2 showed a diffuse pattern throughout the parasite. The second SUN domain protein, TgSLP1, was expressed in a cell cycle-dependent manner and was localised close to the mitotic spindle and, more detailed, at the kinetochore. We demonstrate that conditional knockout of TgSLP1 leads to failure of nuclear division and loss of centrocone integrity.

## Teaser

**Summary:** Identification of a SUN-like protein in the apicomplexan parasite *Toxoplasma gondii* that is essential for replication.

## INTRODUCTION

The phylum Apicomplexa comprises a large number of parasitic protists, including important pathogens, such as *Plasmodium spp.*, the causative agents of malaria, or *Toxoplasma*, a major cause of birth defects and death in immunocompromised patients.

As obligate intracellular parasites, apicomplexans invade host cells in an active process, where they replicate within a parasitophorous vacuole, followed by egress and lysis of the host cell. Similar to what is seen for other apicomplexan parasites, *Toxoplasma gondii* passes through different cellular stages in different hosts. During the tachyzoite stage that exists in the intermediate host, which can be any homeothermic animal, it uses a unique mode of cell division called endodyogeny, where a single round of DNA replication and nuclear mitosis occurs alongside the assembly of two daughter cells within the parental parasite (Francia and Striepen, 2014; White and Suvorova, 2018; Gubbels et al., 2021).

The linker of nucleoskeleton and cytoskeleton (LINC) complex plays critical roles in integrating nuclear and cytoplasmic functions by spanning the nuclear envelope and connecting the cytoskeleton to the nucleus. Therefore, it contributes to multiple important processes, from the transmission of mechanical forces across the nuclear envelope during cell migration to the control of centrosome positioning during DNA replication and repair (Oza et al., 2009; Sato et al., 2009; Katsumata et al., 2017; Horn, 2014; Wang et al., 2018). Its core components are KASH (for ‘Klarsicht, ANC-1 and Syne homology’) domain proteins localised at the outer nuclear membrane and SUN (for ‘Sad1 and UNC-84’) domain proteins, located at the inner nuclear membrane. Both SUN and KASH domain proteins contain transmembrane domains (TMD) and interact with each other in the perinuclear space (Padmakumar et al., 2005; Crisp et al., 2006; Tapley and Starr, 2013). Whereas KASH proteins extend into the cytoplasm where they bind to cytoskeletal proteins, like the actin nucleator formin (Antoku et al., 2015) or microtubule- and actin-interacting proteins (Starr and Han, 2002; Zhen et al., 2002; Patterson et al., 2004; Fridolfsson and Starr, 2010), SUN proteins are anchored in the nucleus through the interaction of nucleoskeletal proteins, such as nuclear lamins (Haque et al., 2006). LINC complexes are highly conserved in eukaryotes and have been identified in mammals (Padmakumar et al., 2005; Crisp et al., 2006; Zhang et al., 2009), *Caenorhabditis elegans* (Malone et al., 1999; Starr et al., 2001), *Drosophila melanogaster* (Patterson et al., 2004), yeasts (Ding et al., 1998; Yamamoto et al., 1999; Jaspersen et al., 2006) and plants (Zhou et al., 2012). Intriguingly, although SUN domain proteins are highly conserved, interacting proteins are often species- or phylum-specific and cannot be identified via standard bioinformatic approaches. For example, the WPP domain proteins in plants show little similarity to known KASH proteins but have been identified as functional analogues (Zhou et al., 2012; Zhou and Meier, 2013; Meier, 2016).

Although LINC complexes can be found in many different species, no cytoskeletal-nuclear bridging complex has been identified in apicomplexan parasites. However, comparable processes exist in apicomplexans, suggesting the existence of a LINC complex. For instance, the apicomplexan parasite *T. gondii* invades host cells through a tight junction that constricts the parasite during penetration. During this process, F-actin accumulates at the posterior pole of the parasite and around the nucleus, suggesting that there is a direct association between F-actin and the nucleus (Del Rosario et al., 2019). This leads to the hypothesis that F-actin and a potential apicomplexan LINC complex are involved in nuclear positioning, protection and deformation during invasion, as observed in other migratory cells (McGregor et al., 2016; Del Rosario et al., 2019). Similarly, the apicomplexan nucleus shows impressive deformations during replication so that it is distributed equally to the forming daughter cells, suggesting the integration of cytoskeletal and nuclear functions (Suvorova et al., 2015).

We identified a protein containing an UNC-50 domain and two SUN domain proteins in the genome of *T. gondii*, that are good candidates to be a core component of the LINC complex. However, no KASH domain proteins could be found using bioinformatic approaches or educated guesses. The UNC-50 domain protein (denoted TgUNC1) was localised to the Golgi and did not seem to be essential for the parasite. Visualisation of one SUN domain protein, TgSLP2 (SUN-like protein 2), showed a diffuse, punctuated pattern through the whole parasite. In contrast, the SUN domain protein TgSLP1 (SUN-like protein 1) demonstrated stage-specific expression, was localised at the mitotic spindle and the centrocone of the parasite and is essential for nuclear division.

## RESULTS

### SUN family proteins in *T. gondii*

**Fig. 1.**
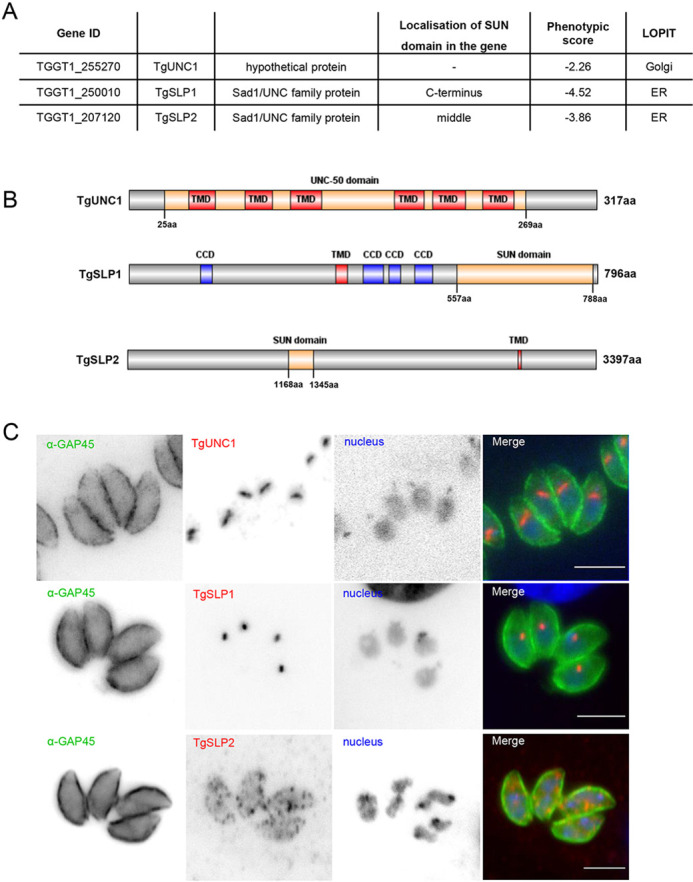
**Overview of Sad1/UNC family proteins in *T. gondii*.** (A) Table of Gene IDs, domain organisation, phenotypic score and prediction of subcellular localisation (LOPIT) of Sad1/UNC family proteins in *T. gondii*. (B) Schematic overview of UNC-50 domain, SUN domain, transmembrane domain (TMD) and coiled-coil domain (CCD) organisation in Sad1/UNC family proteins in *T. gondii*. Numbers indicate the domain position within the protein in amino acids. The figure was created with the IBS Illustrator for Biological Sequences (Liu et al., 2015). (C) Immunofluorescence analysis revealed the localisation of the three UNC family proteins in *T. gondii*. Parasite shape was visualised with the IMC marker anti-GAP45 antibody (α-GAP45), TgUNC1 and TgSLP2 were tagged with 3×HA and were visualised with anti-HA antibody, TgSLP1 has an sYFP2 tag. The nuclei were stained with Hoechst 33342. Images are representative of a minimum of three repeats. Scale bars: 5 µm.

Two proteins that contain a SUN domain and one hypothetical protein containing an UNC-50 domain were identified in the genome of *T. gondii* using the ToxoDB.org database (release 57; Gajria et al., 2008), making them good candidates for being components of an apicomplexan LINC complex. Regarding their domain architecture, they are referred to as TgUNC1 (TGGT1_255270), TgSLP1 for SUN-like protein 1 (TGGT1_250010) and TgSLP2 for SUN-like protein 2 (TGGT1_207120). Based on data resulting from a genome-wide screen of *T. gondii* using CRISPR/Cas9, disruption of *sad1*/*unc* genes caused loss of parasite fitness, indicating that they fulfil crucial roles during the asexual life cycle of the parasite (Fig. 1A; Sidik et al., 2016, 2018). The two SUN domain proteins show a different domain organisation. The SUN domain of TgSLP1 is at the C-terminus, whereas in TgSLP2 it is located in the middle part of the protein (Fig. 1A,B). Both proteins have one predicted transmembrane domain, whereas TgUNC1 possesses multiple predicted transmembrane domains. TgSLP1 has, in addition, multiple coiled-coil regions (Fig. 1B; Jones et al., 2014). Data from a study mapping the subcellular localisation of thousands of proteins in *T. gondii* using a method called hyperplexed localisation of organelle proteins by isotope tagging (hyperLOPIT) shows that TgUNC1 is localised to the Golgi, whereas TgSLP1 and TgSLP2 are most likely localised to the endoplasmic reticulum (ER) (Barylyuk et al., 2020). To assess the localisation of the three UNC family proteins in *T. gondii*, proteins were C-terminally tagged either with a 3×HA epitope tag (TgUNC1 and TgSLP2) or a fluorescent tag (TgSLP1). Whereas TgSLP2 showed a diffuse pattern with multiple foci throughout the cytosol of the parasite, TgUNC1 localised apical to the nucleus, potentially corresponding to the Golgi, and TgSLP1 demonstrated a consistent localisation close to the nucleus (Fig. 1C).

### TgUNC1 localises at the Golgi and is not essential for *T. gondii* growth

Simultaneously with the endogenous labelling of TgUNC1, two LoxP sites, one upstream of the start codon and another downstream of the coding region, were inserted to create a conditional knockout (cKO) line in parasites expressing dimerisable Cre (RH-Δ*ku80-*DiCre; Andenmatten et al., 2013). Correct integration of the tags and efficient Cre-mediated recombination upon the addition of 50 nM rapamycin was confirmed by PCR analysis (Fig. S1A,B). Furthermore, western blot and immunofluorescence analysis demonstrated that the protein is undetectable 48 h post induction (Fig. S1C,E). Further analysis demonstrated that TgUNC1 localised to the Golgi apparatus of the parasite, as seen by colocalisation with the trans-Golgi marker GalNac (Fig. S1E; Nishi et al., 2008) and the cis-Golgi marker GRASP (Fig. S1E; Pfluger et al., 2005). Despite the negative phenotypic score of −2.26 (Sidik et al., 2016), the conditional knockout of *unc1* showed no obvious growth defect, nor any apparent morphological change of the cis- or trans-Golgi in any of the analysed parasites (Fig. S1D,E). Consequently, it was possible to isolate a null mutant of *unc1* after induction of the conditional mutant with rapamycin, that was able to grow normally and presented unaffected organelles such as rhoptries, apicoplast, micronemes, the inner membrane complex or the nucleus (Fig. S1F). In conclusion, TgUNC1 is localised to the Golgi network of the parasite, where it appears to play no critical role for asexual parasite growth and Golgi architecture.

### TgSLP2 shows a diffuse punctuated pattern

We integrated an endogenous tag at the C-terminus of TgSLP2 using CRISPR/Cas9 (Sidik et al., 2016). Despite several attempts, it was only possible to insert a small epitope tag (3×HA). The correct genomic integration of the tag was confirmed by PCR analysis (Fig. S2A,B). In contrast to TgUNC1 and TgSLP1 (see below), the subcellular localisation of TgSLP2 was unclear. It was localised as a diffuse stippled pattern within the parasite and at the intravacuolar network (Fig. S2C). Simultaneous staining of TgSLP2 and the nucleus visualised with a transiently expressed fluorescently labelled histone 2B (H2B; Gubbels et al., 2006) was performed, and it appeared that TgSLP2 is not associated with the nucleus (Fig. S2D). We were interested in how the parasite behaves after the loss of TgSLP2. We tried to integrate a second LoxP site at the 5′ end of *slp2*. Four sgRNAs at different positions to integrate the LoxP site (two upstream of the 5′UTR, one directly upstream of the start codon and one in frame within the coding region of *slp2*) were designed. Despite several transfection attempts, we were unable to generate a parasite line where *slp2* was floxed. As an alternative to the DiCre system, we attempted to use a strategy based on conditional U1 small nuclear ribonucleic particles (snRNP)-mediated gene silencing (Pieperhoff et al., 2015) and another based on the auxin-inducible degron (AID) system (Brown et al., 2017, 2018) to create a conditional knockdown of TgSLP2. However, both methods failed and no integrants could be isolated. It might be possible that our modification strategies interfere not only with the regulation of *slp2,* but also with a potentially important gene, TGGT1_207110 (phenotypic score of −3.68, Sidik et al., 2016), that is just upstream of *slp2.*

### The expression of TgSLP1 is cell cycle dependent, and it colocalises with the mitotic spindle

**Fig. 2.**
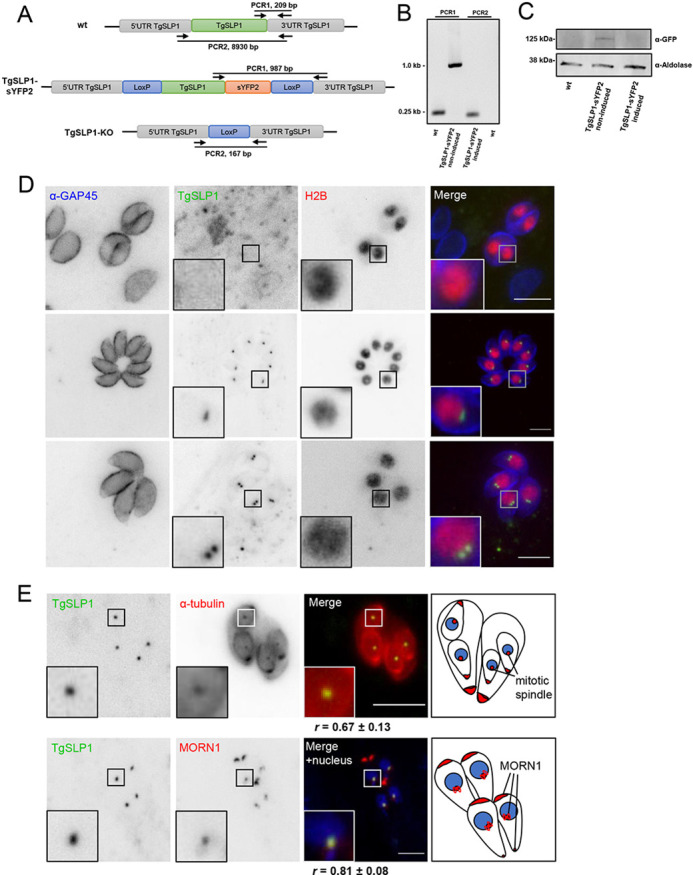
**TgSLP1 colocalises with the mitotic spindle and the centrocone.** (A) Schematic overview of endogenous, sYFP2-tagged and conditional knockout lines of *slp1*. (B) PCR confirmed the correct integration of the tag and excision of *slp1* under induced conditions. Primer positions and length of PCR products are shown. Note that there is no PCR product for the wild-type (wt) PCR2 due to the length of almost 9000 bp. (C) Western blot analysis using α-GFP on the wild-type (wt) and non-induced or induced TgSLP1–sYFP2 lines verified the expected protein size of 88 kDa and protein depletion under induced conditions. α-aldolase was used as loading control. (D) Immunofluorescence analysis of transgenic TgSLP1–sYFP2 parasites and transiently expressed, fluorescently tagged histone 2B (H2B) revealed a close localisation of TgSLP1 to the nucleus. Parasite shape was visualised with anti-GAP45 antibody (α-GAP45). (E) Immunofluorescence analysis revealed that TgSLP1 colocalises with the mitotic spindle and the centrocone, visualised by a transiently expressed, additional copy of fluorescent tagged α-tubulin or MORN1. When indicated, the nuclei were stained with Hoechst 33342. Colocalisation was quantified by calculating the mean±s.d. Pearson correlation coefficient (R) of 20–25 parasites using the ImageJ plugin JACoP as shown under the respective images. Scale bars: 5 µm. All images are representative of a minimum of three repeats.

To determine the localisation of TgSLP1, the C-terminus of *slp1* was endogenously tagged with a yellow fluorescent protein (sYFP2) in RH-Δ*ku80-*DiCre parasites using CRISPR/Cas9. The resulting coding sequence of *slp1-syfp2* was flanked by LoxP sites, allowing the excision of this locus after induction with rapamycin (denote TgSLP1-cKO) (Fig. 2A–C). Genomic integration of the tag and Cre-mediated excision was verified by PCR and depletion of the protein was verified by western blot analysis (Fig. 2B,C).

Interestingly, although a clonal line was obtained, not all parasites expressed TgSLP1–sYFP2 in an asynchronous culture. Whereas some vacuoles had no visible signal, in others TgSLP1 appeared as a single dot or two dots near the nucleus as visualised through a transiently expressed H2B tagged with mRFP (Fig. 2D; Gubbels et al., 2006), leading to the hypothesis that this protein is expressed in a cell cycle-dependent manner as part of the bipartite centrosome of the parasite. The *Toxoplasma* centrosome is divergent from mammalian cells in architecture and composition. For example, the centrioles are composed of nine singlet microtubules, smaller in size than mammalian centrioles (Francia and Striepen, 2014). To label microtubular structures during cell division, TLAP4, a protein previously described to be localised to the cortical microtubules and the centrioles (Liu et al., 2016) was endogenously tagged with mCherry at its N-terminus in TgSLP1–sYFP2 expressing parasites (Fig. S2E,F). Although an overexpressed version of TLAP4 seems to be a good marker for microtubular structures including the mitotic spindle, as previously demonstrated (Liu et al., 2016), the endogenous tagged protein appears to be concentrated at the apical tip of cortical microtubules (Fig. S2G). Therefore, to visualise TgSLP1 in relation to the parasite microtubules, we transiently expressed an extra copy of α-tubulin labelled with mCherry (Hu et al., 2002) in the TgSLP1–sYFP2 parasite line. Analysis of these parasites revealed that TgSLP1 localised close to the mitotic spindle, which is formed during cell division and separates duplicated chromosomes (Fig. 2E, top row).

To provide further subcellular colocalisation, an additional copy of YFP-tagged MORN1 (Gubbels et al., 2006) was transiently transfected into a TgSLP1–mCherry parasite line. MORN1 localises specifically to the apical and posterior end of the inner membrane complex, but also to the centrocone, a specialised nuclear structure that is thought to organise the mitotic spindle and plays a central role in apicoplast segregation and daughter cell formation (Gubbels et al., 2006; Lorestani et al., 2010). As expected, fluorescence microscopy demonstrated close association between the two proteins at the centrocone (Fig. 2E, bottom row).

**Fig. 3.**
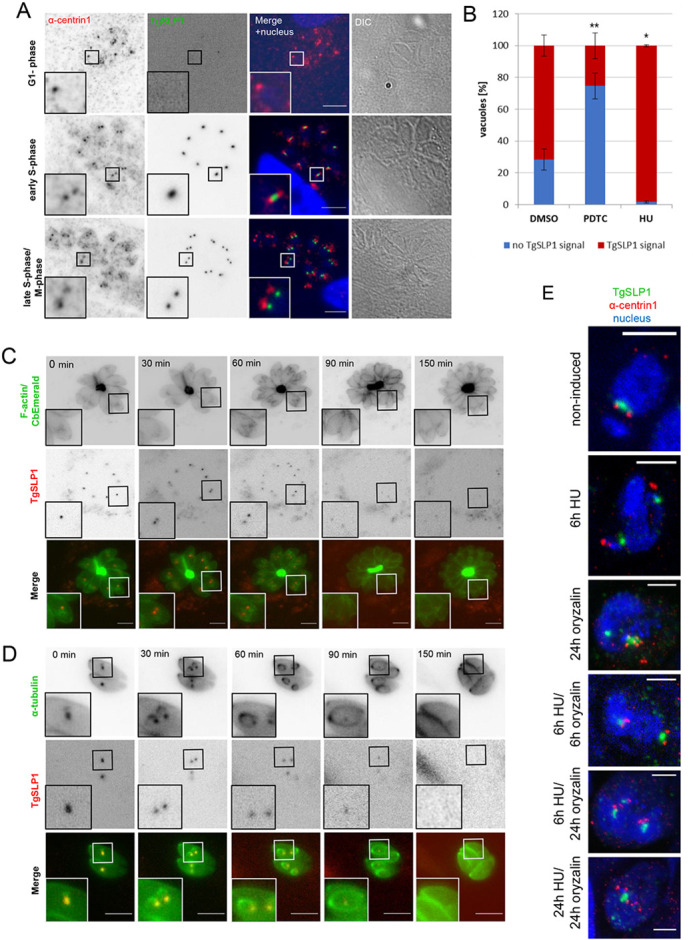
**Dynamic localisation of TgSLP1 throughout the tachyzoite division cycle.** (A) Immunofluorescence analysis throughout the tachyzoite division cycle shows that the expression and localisation of TgSLP1 is cell cycle dependent. Nuclei were stained with Hoechst 33342, the cell cycle stages were defined by determining centrin1 localisation with an anti-centrin1 antibody (α-centrin1). DIC, differential interference contrast. (B) Quantification of TgSLP1 expression in a mixed parasite population (DMSO) and parasites arrested in G1-phase (PDTC) or in S-phase (HU). 100 vacuoles were counted per condition, the experiment was done in biological and technical triplicates. Mean±s.d. values of three independent assays are shown. **P<0.01, *P<0.05 (two-tailed unpaired Student's *t*-test comparing parasites incubated in DMSO versus incubated with PDTC or HU). (C) Time-lapse analysis of TgSLP1 and F-actin localisation during parasite division. TgSLP1 was tagged in a parasite line expressing CbEmerald to visualise F-actin. (D) Time-lapse analysis of TgSLP1 and α-tubulin localisation during parasite division. α-tubulin was visualised through an additional copy of fluorescent tagged α-tubulin in the TgSLP1-sYFP2 parasite line. (E) Treatment with the microtubule polymerisation inhibitor oryzalin did not prevent TgSLP1 localisation near the centrosome in the absence or presence of the cell cycle arresting drug HU, images were made using STED microscopy, centrin1 was visualised with α-centrin1, nuclei were stained with Hoechst 33342. Scale bars: 5 µm (A,C,D), 2 µm (E). All images are representative of a minimum of three biological replicates.

To obtain a better insight into the timing of expression, we used an antibody raised against centrin1 to visualise the centrosome and thus the cell cycle stage of *T. gondii* parasites (Fig. 3A). Tachyzoite endodyogeny is characterised by three phases, consisting of the main phases G1 and S, with mitosis (M-phase) immediately following the completion of DNA replication. With the formation of apical daughter complexes, cytokinesis begins in late S-phase and overlaps with mitosis (Radke et al., 2001). In parasites entering G1-phase with a single centrosome, none or only a very weak signal for TgSLP1 was visible. When cells enter S-phase, the centrosome divides and a single TgSLP1 spot between the divided centrosomes was detectable. This suggests that although TgSLP1 appeared to remain in association with the centrosome, it divides slightly later. Finally, in late S-phase, overlapping with the beginning of M-phase, TgSLP1 divided and showed close association with the centrosome of the parasite (Fig. 3A). To quantify the expression of TgSLP1 in the different phases of cell division, parasites were arrested in G1-phase with pyrrolidine dithiocarbamate (PDTC; Conde de Felipe et al., 2008) or in S-phase using hydroxyurea (HU; De Melo et al., 2000). In an asynchronous population, TgSLP1 was detectable in more than 70% of the vacuoles (Fig. 3B, DMSO; Fig. S3B). In contrast, the majority of vacuoles arrested with PDTC in G1-phase showed no or little TgSLP1 expression (Fig. 3B, PDTC; Fig. S3B). As expected, most of the parasites (more than 98%) arrested in S-phase using HU showed TgSLP1 expression (Fig. 3B, HU; Fig. S3B). Successful arrest was controlled by staining the centrosome (Fig. S3A). The dynamic localisation of TgSLP1 was also observed in live-cell imaging (Movies 1 and 2). Stills from Movies 1 and 2 depicting different stages of the division cycle of the parasite are shown in Fig. 3C and D. TgSLP1 was tagged with mCherry in parasites expressing an F-actin-binding chromobody tagged with emerald (CbEmerald; Fig. 3C; Periz et al., 2017). Fig. 3D shows cell division in parasites transiently expressing mCherry tagged α-tubulin (Hu et al., 2002). The formation of daughter parasites within the mother demonstrates the onset of cytokinesis, concomitant with TgSLP1 division (Fig. 3C,D; 30–60 min). TgSLP1 disappeared just before daughter cells emerged from the mother cell (Fig. 3C,D; 90–150 min).

Interestingly, the localisation of TgSLP1 does not depend on mitotic spindle formation given that treatment with oryzalin, in the presence or absence of the cell cycle arresting drug HU, did not prevent the localisation of this protein near the centrosomes (Fig. 3E; Fig. S3C).

### TgSLP1 is essential for the tachyzoite division cycle

**Fig. 4.**
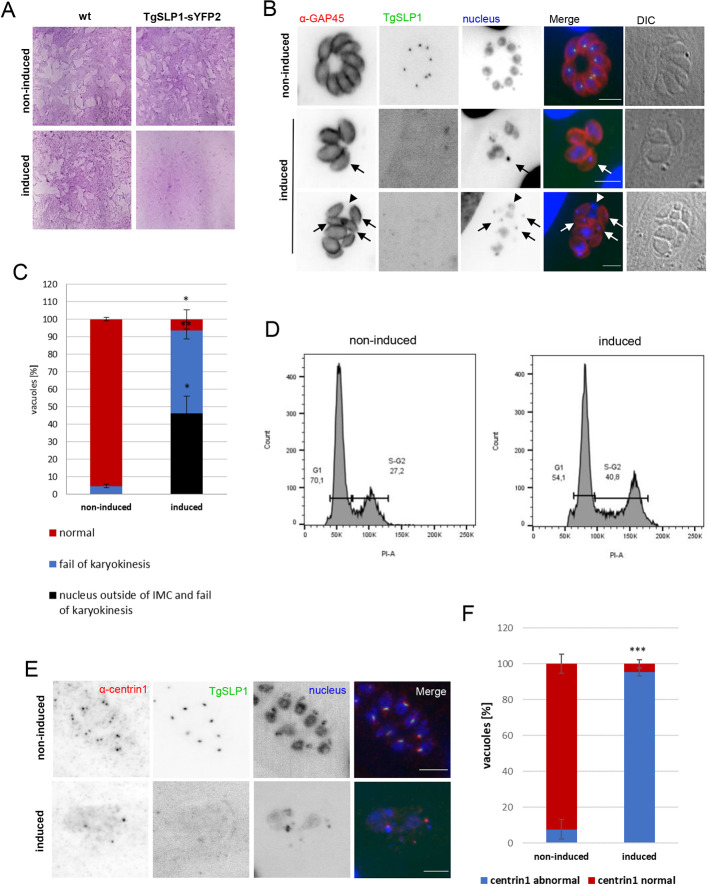
**Parasites lacking TgSLP1 are not viable due to severe nuclear defects.** (A) A plaque assay showed that loss of TgSLP1 is strongly affecting parasites growth. Images represent an ∼2 cm width. (B) Immunofluorescence analysis of the TgSLP1 conditional knockout line non-induced or induced. TgSLP1 cannot be detected under induced conditions. Arrows mark parasites without a nucleus; arrowheads mark a nucleus outside of the parasite. Parasite shape was visualised with anti-GAP45 antibody (α-GAP45) and the nuclei were stained with Hoechst 33342. DIC, differential interference contrast. Scale bars: 5 µm. Images in A and B are representative of three biological replicates. (C) Quantification of vacuoles with nuclear loss and thus failed karyokinesis and vacuoles with additional nuclear DNA outside of the IMC. 100 vacuoles were counted each under induced (48 h rapamycin) and non-induced (DMSO) conditions. The experiment was done in biological triplicates. Error bars indicate the s.d. **P<0.01, *P<0.05 (two-tailed unpaired Student's *t*-test comparing parasites incubated in DMSO versus incubated with rapamycin). (D) FACS analysis of DNA content in non-induced and induced TgSLP1–sYFP2 parasites reveals an increase of DNA content in induced parasites. Numbers represent percentages at each stage. (E) Immunofluorescence analysis of the TgSLP1 conditional knockout line under non-induced or induced conditions. The centrosome of the parasite was visualised with an anti-centrin1 antibody (α-centrin1), and the nuclei were stained with Hoechst 33342. Scale bars: 5 µm. (F) Quantification of vacuoles with normal centrin1 signal and abnormal centrin1 signal. 100 vacuoles were counted each under induced (48 h rapamycin) and non-induced (DMSO) conditions. The experiment was done in biological triplicates. Error bars indicate the s.d. ***P<0.001 (two-tailed unpaired Student's *t*-test comparing parasites incubated in DMSO versus incubated with rapamycin).

To investigate the role of TgSLP1 in *T. gondii*, the conditional knockout line (TgSLP1-cKO) was induced with 50 nM rapamycin. The viability of the conditional TgSLP1–sYFP2 parasites was tested with a plaque assay and no growth was detectable 7 days post induction (Fig. 4A), confirming the fitness score of −4.52 (Sidik et al., 2016). The growth of wild-type parasites is not affected by the addition of rapamycin (Fig. 4A, induced), as previously reported by Andenmatten et al. (2013). Phenotypic analysis of individual *T. gondii* vacuoles was performed using an immunofluorescence assay. Although significantly reduced TgSLP1 expression was detectable up to 24 h after addition of rapamycin, no TgSLP1 signal was detectable after 48 h of induction (Fig. S3D). In the absence of TgSLP1, parasites were deformed, and the nucleus failed to divide (Fig. 4B). Nearly all vacuoles showed a strong defect in karyokinesis (Fig. 4C). Whereas some parasites from one vacuole harboured an extremely enlarged nucleus, others lacked nuclear DNA and only possessed DNA from the apicoplast. This phenotype was observed in more than 90% of the vacuoles 48 h after induction. Almost 50% of the vacuoles showed additional, severe nuclear segregation defects, where nuclear DNA appeared outside of the inner membrane complex (IMC). To determine whether this defect was due to a defect in nuclear division or failure of DNA replication, we performed FACS analysis of DNA content (Fig. 4D; Fig. S4). In the initial forward versus side scatter gating (FSC versus SSC), we could observe differences in size and granulosity between the induced and non-induced populations (Fig. S4B) corroborating the observation of parasites presenting aberrant morphology after addition of rapamycin (Fig. 4B). TgSLP1-cKO clearly shows a shifting of the normal cell division curves to the right (Fig. 4D) showing nuclei with higher DNA content and arrested in mitosis (with an average of 40.5% in the induced parasites versus 26.7% in the non-induced). In good agreement, deletion of TgSLP1 resulted in diffuse localisation and/or loss of the centrosome at individual nuclei within a parasitophorous vacuole, indicating that TgSLP1 is a crucial part of the centrosome, required for its integrity (Fig. 4E,F).

### STED analysis demonstrates TgSLP1 localisation to the kinetochore

**Fig. 5.**
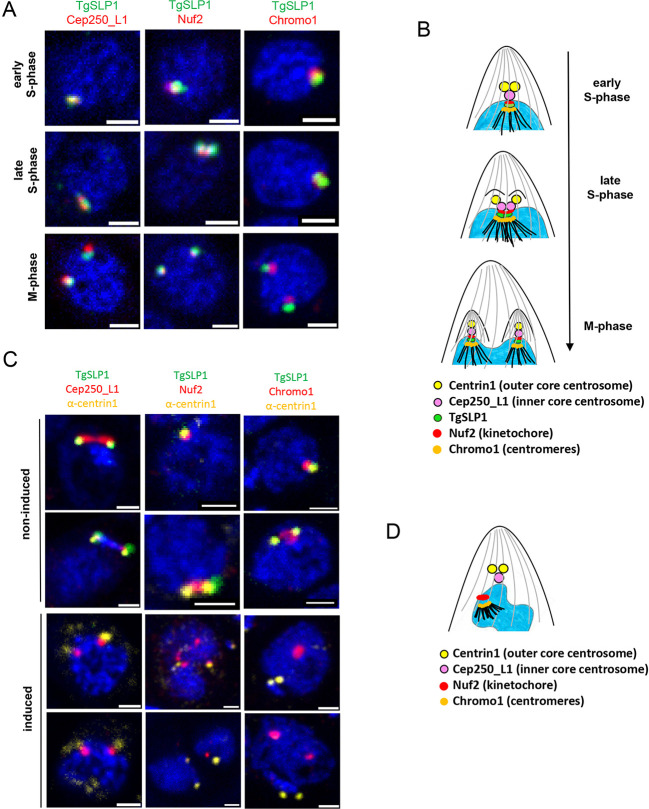
**TgSLP1 localises at the kinetochore and seems to link the centromeres to the nuclear envelope during cell division.** (A) 3D-STED super-resolution microscopy of parasite nuclei that are in different cell cycle stages expressing TgSLP1–sYFP2 and Cep250_L1–3×HA (inner core centrosome), Nuf2–3×HA (kinetochore) or Chromo1–3×HA (centromeres). Nuclei were stained with Hoechst 33342. Scale bars: 1 µm. (B) Schematic summary of the *T. gondii* centrosome (outer core: centrin1, yellow; inner core: Cep250_L1, purple), kinetochore (Nuf2, red) and centromeres (Chromo1, orange) in regard to TgSLP1 (green) in different stages of the cell cycle. (C) 3D-STED super-resolution microscopy of parasite nuclei expressing Cep250_L1–3×HA, Nuf2–3×HA or Chromo1–3×HA in TgSLP1-cKO parasites. Centrin1 was visualised with an anti-centrin1 antibody (α-centrin1), and the nuclei were stained with Hoechst 33342. Scale bars: 1 µm. (D) Schematic representation of centrosome (outer core: centrin1, yellow; inner core: Cep250_L1, purple), kinetochore (Nuf2, red) and centromeres (Chromo1, orange) in TgSLP1-cKO parasites. All images are representative of a minimum of three biological replicates.

Previous studies have defined the morphological changes of the parasite centrosome during mitosis and have demonstrated that the outer core divides prior to the inner core, which in turn divides prior to the centromeres (Suvorova et al., 2015; Tomasina et al., 2022). Given that TgSLP1 appears to divide after division of the outer core, as seen by colocalisation with the outer core marker centrin1 (Fig. 3A), we performed an analogous localisation analysis with additional markers of the centrosome, such as Cep250_L1 (inner core centrosome; Suvorova et al., 2015), Nuf2 (kinetochore; Farrell and Gubbels, 2014) and Chromo1 (centromeres; Gissot et al., 2012). Each of these proteins was endogenously tagged with 3×HA and colocalisation analysis was performed using 3D-STED super-resolution microscopy (Fig. 5A). Collectively these data demonstrate that TgSLP1 divides after Cep250_L1, at the same time as Nuf2, but prior to Chromo1, establishing that TgSLP1 divides at the same time as the kinetochore (Fig. 5A,B). Indeed, recent studies have demonstrated that the LINC complex, in particular SUN proteins, can be required for kinetochore clustering (Yadav and Sanyal, 2018). To obtain a better overview of centrosome defects, we performed 3D-STED analysis of parasites expressing tagged versions of Cep250_L1, Nuf2 and Chromo1 grown in presence and absence of rapamycin (Fig. 5C).

Whereas, in non-induced parasites, all markers appeared tightly linked (Fig. 5C), absence of TgSLP1 led to the incorrect localisation and division of all markers analysed here (Fig. S3E). Given that the individual markers seem to be distributed randomly within the nucleus (Fig. S3E), we performed colocalisation analysis with centrin1 and Cep250_L1, Nuf2 or Chromo1, respectively in the absence of TgSLP1. Looking at centrin1 and Cep250_L1, it seems that the outer core and inner core of the centrosome are still associated, and their interaction is unaffected by the loss of TgSLP1 (Fig. 5C left panel). In contrast, the kinetochore and centromeres (Nuf2 and Chromo1) show aberrant localisation with respect to the centrosome (centrin1), and it appears that the connection between the centrosome and the kinetochore or centromeres is lost in induced parasites (Fig. 5C, middle and right panels). Based on this analysis, we suggest that TgSLP1 might be the linker between the centrosome and the kinetochore/centromeres (Fig. 5D).

In conclusion, the severe defects of nuclear division resulting from deletion of TgSLP1 are in good agreement with its role as a protein of the LINC complex and suggest that there is an interaction of the nucleus with the mitotic spindle.

## DISCUSSION

The connection between the nucleus and the cytoskeleton is central for the maintenance of a variety of cellular processes, including attachment of the centrosome to the nucleus during cell division. In metazoans, plants and single-cell organisms like yeast, the cytoskeletal–nuclear bridge involves the LINC complex, which comprises SUN domain proteins and KASH domain proteins (Padmakumar et al., 2005; Crisp et al., 2006). We identified the two SUN domain proteins TgSLP1 and TgSLP2, and the UNC-50 domain protein TgUNC1 in the genome of the apicomplexan parasite *T. gondii*. Whereas the non-essential UNC-50 protein TgUNC1 localised to the Golgi, the mid-SUN domain protein TgSLP2 localised as a diffused punctuated pattern through the parasite. Mid-SUN proteins have been characterised in *Arabidopsis thaliana* (AtSUN3 and AtSUN4; Graumann et al., 2014) and in mice (Opt; Sohaskey et al., 2010). AtSUN3 and AtSUN4 localise to the nuclear envelope and the ER. In *A. thaliana*, both C-terminal SUN and mid-SUN domain proteins have been shown to interact with each other, as well as with the KASH domain protein AtWIP1, and are involved in a protein complex network at the nuclear envelope that is reminiscent of the LINC complex found in other species (Graumann et al., 2014). In mice, the mid-SUN protein Opt is localised to the ER and might act as an adaptor protein connecting the rough ER to the cytoskeleton (Sohaskey et al., 2010). The subcellular localisation prediction from the LOPIT study suggests that TgSLP2 localises at the ER (Barylyuk et al., 2020). Based on the observations in the immunofluorescence assays, the *T. gondii* mid-SUN protein TgSLP2 might partially colocalise with the ER. Although we were unable to generate a conditional knockout mutant of TgSLP2, the negative phenotypic score of −3.86 suggested from the CRISPR/Cas9 genome-wide screen (Sidik et al., 2016) indicates that TgSLP2 is essential. However, there is too little information to definitively exclude TgSLP2 as being part of a nuclear-cytoskeletal bridging complex.

In this study, we show that TgSLP1 is a member of the SUN domain family in the apicomplexan parasite *T. gondii* and provide evidence that it is essential for cell division in the tachyzoite stage. TgSLP1 localises at the mitotic spindle, specifically at the kinetochore, and seems to be closely associated to the centrosome during the asexual division cycle of *T. gondii*. Parasites lacking TgSLP1 show a severe defect in centrosome integrity and nuclear segregation (Figs 4 and 5).

Studies in mammals (Zhang et al., 2009), *C. elegans* (Malone et al., 2003; Zhou et al., 2009) and yeast (Chen et al., 2019) have shown that non-canonical LINC complexes connect the centrosome to the nucleus. For example, the SUN domain proteins SUN1 and SUN2 form complexes with the KASH domain protein syne-2 (also known as nesprin-2) to couple the nucleus to the centrosome during neurogenesis and neuronal migration in mice (Zhang et al., 2009). In the nematode *C. elegans*, the centrosome is attached to the nucleus through the linkage of the SUN-KASH pair SUN-1 and ZYG-12 (Malone et al., 2003; Zhou et al., 2009). A recent study in budding yeast has revealed an atypical centrosome-associated LINC complex formed by the SUN protein Mps3 and the KASH-like protein Mps2 during mitosis (Chen et al., 2019).

Our results on TgSLP1 are in good agreement with the function of SUN domain proteins described in *C. elegans* and yeast. Whereas in *sun-1*-depleted worms, centrosomes were detached from the nucleus (Malone et al., 2003), it has been shown that yeast Mps3 is required for the duplication of the spindle pole body (Jaspersen et al., 2006). Our findings allow us to conclude that TgSLP1 might be part of an apicomplexan LINC complex linking centromeres to the centrosome and therefore to the nuclear envelope. Nevertheless, we still lack a KASH-like protein as a binding partner of TgSLP1. Interestingly, other centrosome-associated LINC complexes have SUN-binding partners that differ from the typical KASH domain proteins. For instance, *C. elegans* ZYG-12 has three isoforms, two at the nuclear envelope, both of which harbour a transmembrane-containing KASH domain, and one, localising at the centrosome, which is missing the KASH domain (Zhou et al., 2009). Furthermore, the yeast KASH-like protein Mps2 lacks the typical C-terminal KASH motif, but it interacts with Mps3 similar to conserved SUN-KASH binding. Given that we were unable to identify KASH domain proteins with standard bioinformatic approaches in *T. gondii*, it seems likely that the SUN-binding counterpart is an atypical KASH-like protein, similar to what has been observed in other organisms. In an attempt to identify potential interaction partners of TgSLP1, we used proximity labelling (TurboID; Branon et al., 2018; Zhou et al., 2021), but due to inconsistent results, no KASH-like protein could be identified using this method (data not shown). Based on an educated guess, we studied two hypothetical proteins (TGGT1_279360 and TGGT1_321410) with negative phenotypic scores (Sidik et al., 2016) for their subcellular localisation. TGGT1_279360 contains a PPPX motif that is essential for binding the SUN domain (Padmakumar et al., 2005) and the suggested subcellular localisation of TGGT1_321410 is, depending on the prediction algorithm, in the nucleus or at the plasma membrane (Barylyuk et al., 2020), making them interesting candidates for being KASH-like proteins. Although both proteins appeared to localise near the nucleus, they were not consistently in close association with TgSLP1 and are probably localised to the Golgi (data not shown). In conclusion, we identified the SUN domain protein TgSLP1 as being required for centrocone integrity and proper nuclear segregation during endodyogeny. We suggest that TgSLP1 is part of an apicomplexan-specific LINC complex that connects the bipartite centrosome to the centromeres, although a KASH-like binding partner remains to be discovered.

## MATERIALS AND METHODS

### Cultivation of *T. gondii* and host cells

*T. gondii* tachyzoites of RH-Δ*ku80-*DiCre (Andenmatten et al., 2013) and the resulting parasite lines generated in this study were maintained in confluent human foreskin fibroblasts (HFFs; LGC/ATCC SCRC-1041) cultured at 37°C and 5% CO_2_ in Dulbecco's modified Eagle's medium (DMEM; Sigma D6546) supplemented with 10% fetal bovine serum (FBS; BioSell FBS.US.0500), 4 mM L-glutamine (Sigma G7513) and 25 µg ml^−1^ gentamycin (Sigma G1397).

### Transfection and selection of *T. gondii* tachyzoites

To generate stable parasite lines, parasites were transfected using a P3 Primary cell 4D-Nucleofector X kit L, V4XP-3024 from Lonza. 10^6^–10^7^ freshly lysed RH-Δ*ku80-*DiCre parasites were centrifuged (1500 ***g*** for 5 min), resuspended in 100 µl P3 buffer, mixed with prepared DNA (see section, ‘Generation of tagged and floxed strains’) and electroporated within a 100 µl cuvette using the Amaxa 4D-Nucleofector system from Lonza. For electroporation, the programme FI-158 was used. Transfected parasites were resuspended in fresh DMEM and added onto confluent HFF cells.

To transiently transfect parasites, 10^6^–10^7^ freshly lysed parasites of the respective strain were centrifuged, resuspended in 100 µl P3 buffer and mixed with 5–10 ng of ethanol-precipitated plasmid DNA. Parasites were electroporated as described above and transferred to HFF cells grown on coverslips in 24-well-plates. When indicated, parasites were induced directly by the addition of 50 nM rapamycin (Sigma R0395). An equal amount of dimethylsulfoxide (DMSO; Roth 4720.4) was added to all controls (non-induced). After 24–48 h, transiently transfected parasites were fixed and prepared for microscopy (see section ‘Immunofluorescence assay’).

### Generation of tagged and floxed strains

To endogenously tag genes in *T. gondii,* genome editing via CRISPR/Cas9 described by Stortz et al. (2019) was used. To generate sgRNA vectors for specific cleavage of DNA, sgRNAs were designed using the EuPaGDT software (Table S1; Peng and Tarleton, 2015) and cloned into a pU6-DHFR vector coding for nuclear Cas9-YFP expression (Tub-Cas9-YFP-pU6-ccdB-tracrRNA; Curt-Varesano et al., 2016) via endonuclease digestion with BsaI-HFv2 (New England Biolabs R3733S), primer annealing and standard ligation as previously described (Curt-Varesano et al., 2016). The repair templates for the C- or N-terminal tags (3×HA, mCherry, sYFP2) were generated by PCR using Q5 DNA polymerase (New England Biolabs M0491S) and oligonucleotides with 50 bp of flanking homologies. Repair templates for the upstream LoxP sequence were ordered as oligonucleotides being flanked with 33 bp homology sequences on each side (Stortz et al., 2019). To generate tagged or floxed strains, PCR products, purified with the extractme DNA clean-up kit from Blirt (EM26.1-250) or 10 µM oligonucleotide (for LoxP integration) were mixed with 10 ng of the Cas9–YFP plasmid containing the respective sgRNA sequence, ethanol-precipitated, pelleted (15,000 ***g*** for 30 min at 4°C) and used immediately for transfection. Parasites were incubated for 24–48 h on HFF cells, mechanically released from the host cells, filtered and sorted using fluorescence-activated cell sorting (FACS) for transient nuclear Cas9–YFP expression (FACSAria III Cell Sorter, BD Biosciences) into 96-well-plates containing confluent HFF cells (5–10 YFP expressing parasites/well). After 5–7 days of incubation, plates were screened for parasite plaques, parasite DNA was isolated using the extractme genomic DNA kit from Blirt (EM13-250) and the integration of the repair template was confirmed by PCR and sequencing (Eurofins Genomics). To validate band sizes in agarose gels, 1 kb plus DNA ladder (New England Biolabs N3200S) or 250 bp DNA molecular weight marker XVI (Roche 11855638001) was used. All oligonucleotides used in this study were ordered from Thermo Fisher Scientific and are listed in Table S2.

### Western blotting

For protein detection via immunoblot analysis, parasites were cultured for 72 h on HFFs and induced with 50 nM rapamycin 24 h post infection if indicated. 10^6^ parasites per line and condition were pelleted at 4°C and 1500 ***g*** for 5 min, washed in cold phosphate-buffered saline (PBS; Sigma D8537), briefly frozen at −80°C, mixed with 10 µl of Orange loading dye (125 mM Tris-HCl pH 6.5; 50% glycerol; 4% SDS; 0.2% Orange G, Sigma O3756) and 0.1 M dithiothreitol (DTT; Sigma D0632), boiled at 100°C for 10 min and loaded onto a 4–20% precast polyacrylamide mini gel (Bio-Rad 4561094). For labelling, antibodies against GFP or HA and as loading control an antibody against *T. gondii* aldolase was used. Chameleon Duo pre-stained protein ladder (Li-Cor 928-60000) was used for size validation. Stained membranes were imaged using Odyssey CLX (Li-Cor). All antibodies used in this study are listed in Table S3. Images of uncropped plots for figure in this paper are shown in Fig. S5.

### Immunofluorescence assay

For immunofluorescence analysis, infected HFF monolayers grown on coverslips were fixed for 30 min using 4% paraformaldehyde (PFA; Science services E15713) in PBS at room temperature. Samples were blocked and permeabilised for 30 min using 2% bovine serum albumin (BSA; Sigma A7030) and 0.2% Triton X-100 (Sigma T8787) in PBS. Antibody labelling was performed using the indicated combinations of primary antibodies for 1 h, followed by incubation with secondary antibodies for an additional hour. When indicated, the nucleus was stained with 1 µg ml^−1^ Hoechst 33342 (Thermo Fisher Scientific 62249). Three washes with PBS were performed between antibody incubations. Samples were mounted with ProLong Gold Antifade Mountant (Thermo Fisher Scientific P36934). The antibodies used in this study are listed in Table S3. In Fig. S1, the naturally biotinylated apicoplast was stained with a streptavidin conjugate (streptavidin–Alexa Fluor 594 conjugate, Invitrogen S11227).

### Plaque assay

Growth assays were performed in a six-well-plate; HFF cells were infected with 1000 parasites per well and incubated for 7 days either with 50 nM rapamycin (induced) or DMSO (non-induced) and stained with Giemsa solution (Roth T862.1) to visualise plaques.

### Imaging

All widefield microscopy images and movies were acquired on a Leica DMi8 widefield microscope with the Leica Application Suite X (Las X) and processed with Fiji software (Schindelin et al., 2012). Time-lapse video microscopy was performed using glass bottom dishes in a closed chamber to maintain culture conditions. The Pearson correlation coefficient R of 20–25 parasites was calculated using the ImageJ plugin JACoP (Bolte and Cordelières, 2006).

The super-resolution microscopy images of parasite nuclei were acquired on a Abberior 3D-STED microscope using the confocal setting for Hoechst 33342 and STED setting for centrin1, TgSLP1, Cep250_L1, Nuf2 and Chromo1 imaging.

### Cell cycle arresting and microtubules depolymerisation drugs

Parasites were arrested in G1-phase using 80 µM pyrrolidine dithiocarbamate (PDTC; Sigma P8765) or in S-phase using 300 µM hydroxyurea (HU; Sigma H8627). TgSLP1–sYFP2 parasites were preincubated on coverslips in 24-well-plates without drugs and after 24 h treated with PDTC, HU or DMSO as control. After 6 h, cells were fixed with 4% PFA followed by immunofluorescence assay. 100 vacuoles per condition were counted for TgSLP1 expression. The experiment was carried out in biological and technical triplicates.

To verify whether microtubules were important for the localisation of TgSLP1 to the centrosome, parasites were incubated with 2.5 µM Oryzalin (Sigma 36182) for 6 h of 24 h in the presence or absence of 300 µM HU.

### FACS DNA content analysis

HFFs were infected with TgSLP1–sYFP2 parasites in the presence or absence of 50 nM rapamycin for 48 h prior to collection. Parasites were mechanically released from the host cells, filtered through a 3 µm membrane and pelleted at 1200 ***g*** for 5 min. Pellets were resuspended in 300 µl of cold PBS. 700 µl of ice-cold 100% ethanol was added to the parasites drop by drop. Parasites were stored at −20°C until analysis.

On the day of the analysis, parasites were centrifuged for 5 min at 1500 ***g*** and washed twice in PBS. Afterwards, parasites were incubated at room temperature for 30 min in 1 ml of PBS with 20 µl of RNase (Blirt) and propidium iodide (PI, Applichem A2261) to a final concentration of 0.2 mg ml^−1^. To remove large debris, parasites were filtered through a 30 µm filter before analysis on a FACSCanto II (BD Biosciences). The speed of the flow was adjusted to around 400 events/s. Doublets were excluded by PI-height versus PI-width gating and at least 20,000 single events per sample were acquired. Cell cycle analysis was performed using FlowJo^TM^ software. A summary of the gating strategy is shown in Fig. S4A. This analysis was carried out in triplicates.

## Supplementary Material

10.1242/joces.260337_sup1 Supplementary information
