# Supplementary material for: The SUN-like protein TgSLP1 is essential for nuclear division in the apicomplexan parasite Toxoplasma gondii
Source: J Cell Sci. 2023 Oct 30;136(21):jcs260337. doi: 10.1242/jcs.260337 (PMC10629696; doi:10.1242/jcs.260337)
Supplement: Supplementary information [file joces-136-260337-s1.pdf]

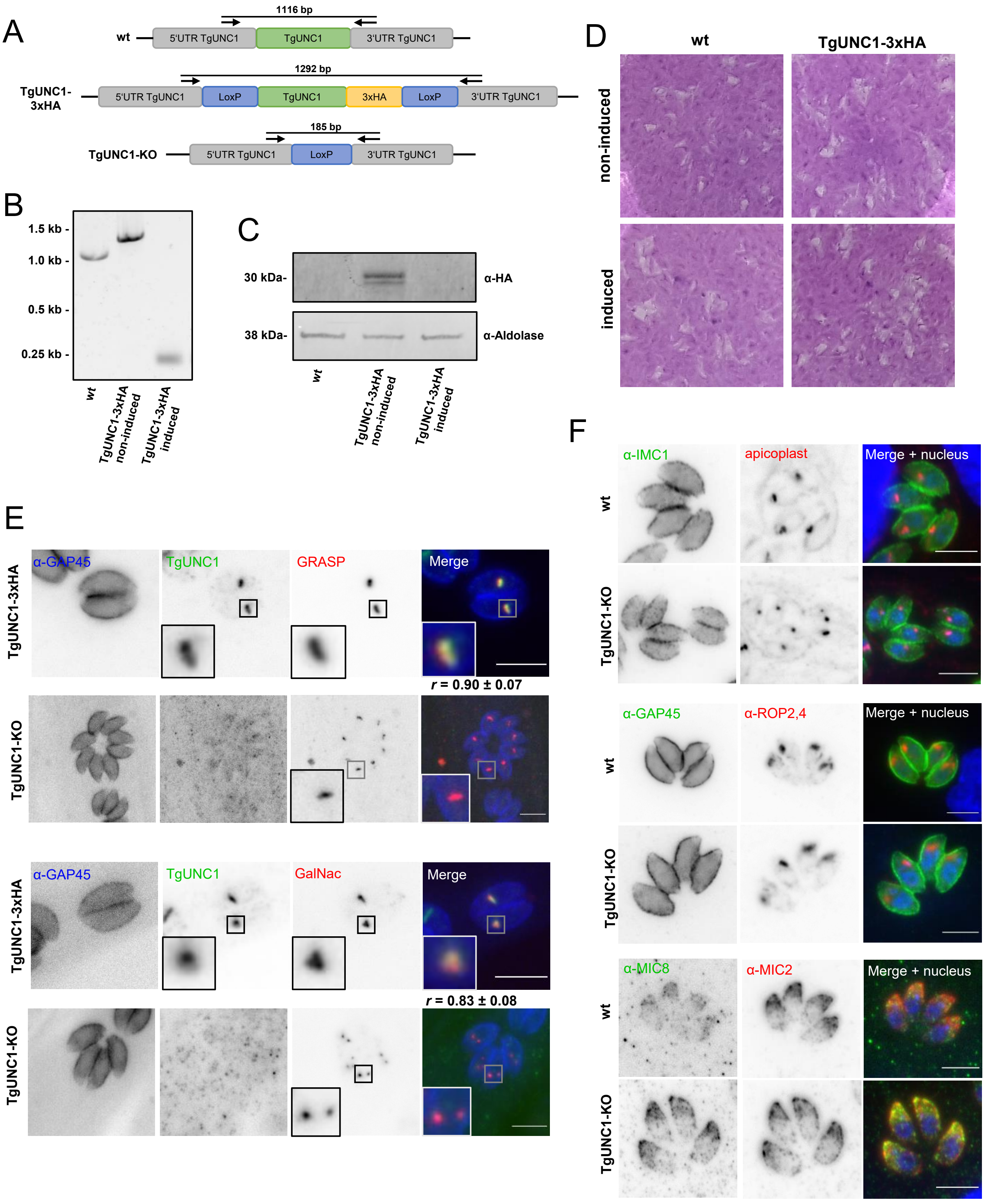

Journal of Cell Science • Supplementary information

Fig. S1. TgUNC1 localises to the Golgi apparatus and is not required for parasite growth. (A) Schematic overview of endogenous, 3xHA tagged and conditional knockout lines of unc1. (B) PCR analysis confirmed the correct integration of tags and excision of unc1 under induced conditions. Primer positions and length of PCR products are shown in (A). (C) Western blot analysis using α-HA on the wildtype (wt) and non-induced or induced TgUNC1-3xHA lines verified the expected protein size of around 35 kDa and protein depletion under induced conditions. α-Aldolase was used as loading control. (D) Plaque assay showed that loss of TgUNC1 is not affecting the parasite's growth. (E) Immunofluorescence analysis revealed that TgUNC1 colocalises with the Golgi apparatus. The colocalisation was quantified by calculating the Pearson correlation coefficient (R) of 20-25 parasites using the ImageJ plugin JACoP. Mean values and standard deviation are shown under the respective images. Immunofluorescence analysis of the TgUNC1-3xHA line or an isolated knockout mutant of TgUNC1 (TgUNC1-KO) showed normal shape of the parasites and the Golgi. In the knockout line, TgUNC1-3xHA cannot be detected with α-HA. Parasite shape was visualised with α-GAP45, GRASP (cis-Golgi) and GalNac (trans-Golgi) with a transiently expressed copy of GRASP or GalNac labelled with a fluorescent tag. (E) Immunofluorescence analysis of different marker proteins on the wildtype (wt) and an isolated unc1 null mutant (TgUNC1-KO) showed normal shape of all organelles analysed in this experiment. The shape of the parasites was visualised with α-GAP45 or α-IMC1, rhoptries with α-ROP2,4, micronemes with α-MIC2 or α-MIC8 and the naturally biotinylated apicoplast with a streptavidin conjugate. The nuclei were stained with Hoechst. Scale bar: 5 µm.

Journal of Cell Science • Supplementary information

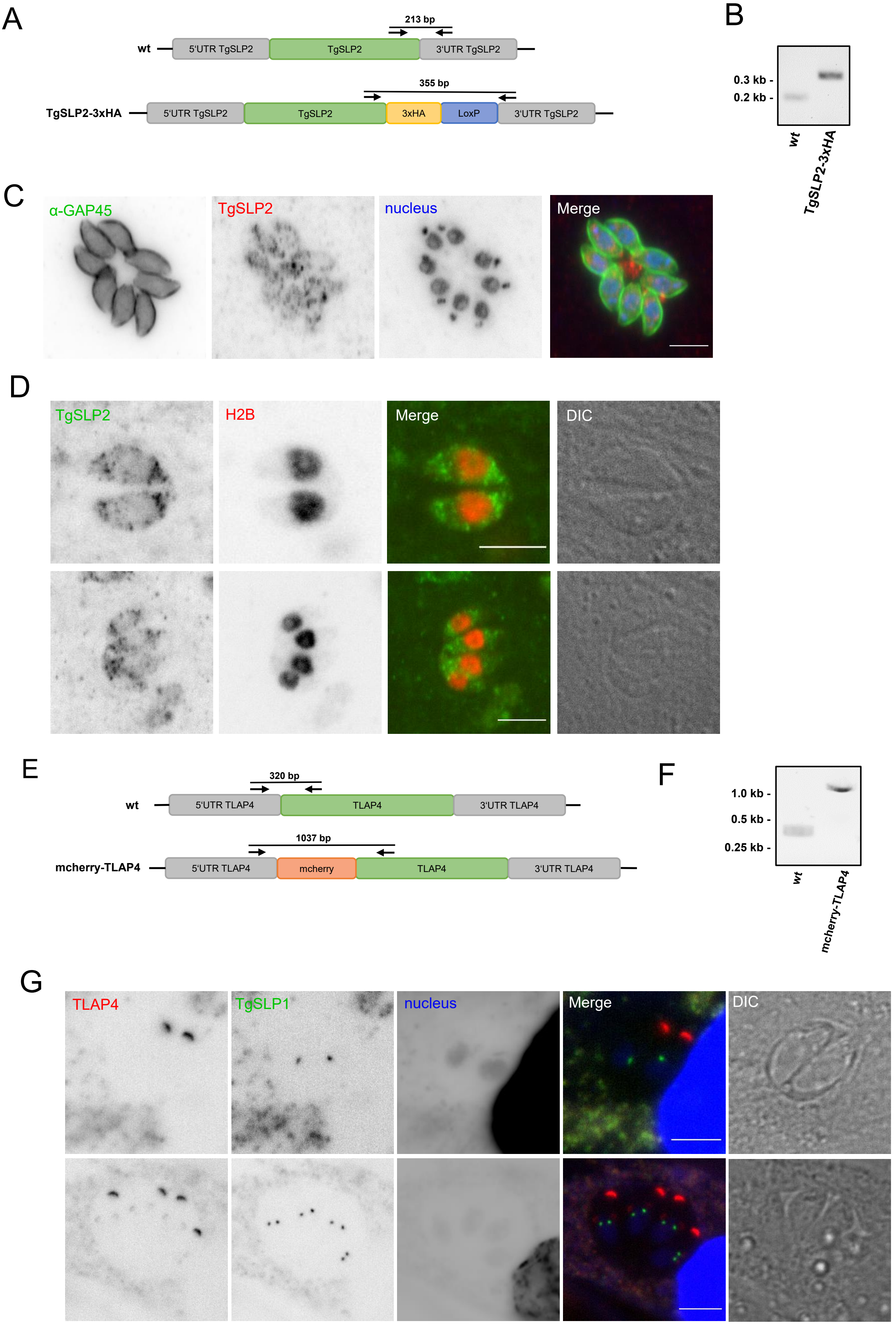

Journal of Cell Science • Supplementary information

Fig. S2. TgSLP2 localises as a diffuse stippled pattern through the parasite and TLAP4 is localised at the apical tip of mother and daughter parasites (A) Schematic overview of endogenous and C-terminal tagged lines of TgSLP2. (B) PCR confirmed the correct integration of tag. Primer positions and length of PCR products are shown in (A). (C) Immunofluorescence analysis of transgenic TgSLP2-3xHA parasites, TgSLP2 was visualised with α-HA, the parasite shape was visualised with α-GAP45 and the nuclei were stained with Hoechst. (D) Simultaneous staining of TgSLP2 with α-HA and the nucleus with a transiently expressed H2B-mRFP. DIC: differential interference contrast, scale bar: 5 µm. (E) Schematic overview of wildtype (wt) and the N-terminally, mCherry-tagged parasite line of TLAP4. (F) PCR analysis confirmed the correct integration of the tag. Primer positions and length of PCR products are shown in (E). (G) Immunofluorescence analysis of C-terminal tagged TgSLP1-sYFP2 and N-terminal tagged TLAP4-mCherry parasites. The nuclei were stained with Hoechst. DIC: differential interference contrast, scale bar: 5 µm

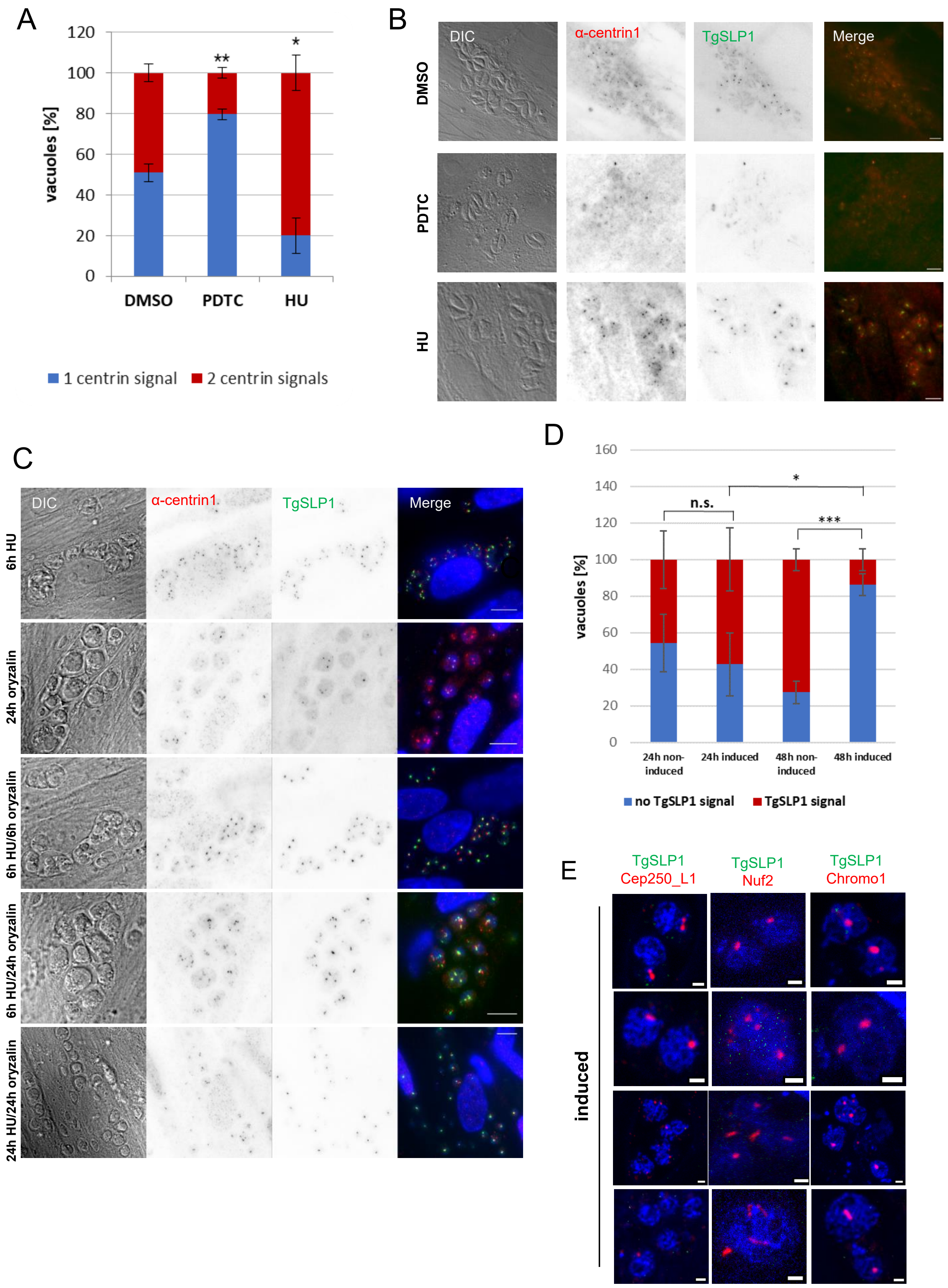

Journal of Cell Science • Supplementary information

Fig. S3. Quantification and representative images of cell cycle arresting drugs HU and PDTC and the microtubule polymerisation inhibitor oryzalin. (A) Quantification of α-centrin1 signals in a mixed parasite population (DMSO) and parasites arrested in G1 phase (PDTC) or in S-phase (HU). 100 vacuoles were counted per condition, the experiment was done in biological and technical triplicates. ** $p<0.01$, * $p<0.05$ obtained in a two-tailed student t-test comparing parasites incubated in DMSO vs incubated with PDTC or HU. Mean values of three independent assays are shown, error bars indicate the standard deviation. (B) Representative immunofluorescence images of the cell cycle arrest experiment. The centrosome of the parasite was visualised with α-centrin1, TgSLP1 was tagged with sYFP2. DIC: differential interference contrast, scale bar: 5µm. (C) Representative immunofluorescence images of whole vacuoles treated with the microtubule polymerisation inhibitor oryzalin. TgSLP1 localisation near the centrosome in the absence or presence of the cell cycle arresting drug hydroxyurea (HU) was not affected. (D) Quantification of TgSLP1 expression after 24 hours or 48 hours treatment with rapamycin. 70-100 vacuoles were counted per condition, the experiment was done in biological triplicates. *** $p<0.001$, * $p<0.05$, n.s. not significant obtained in a two-tailed student t-test. Mean values of three independent assays are shown, error bars indicate the standard deviation. (E) Representative 3D-STED microscopy images of the inner core centrosome (Cep250_L1), the kinetochore (Nuf2) and the centromeres (Chromo1) in TgSLP1-KO parasites, scale bars: 1 µm.

Journal of Cell Science • Supplementary information

A

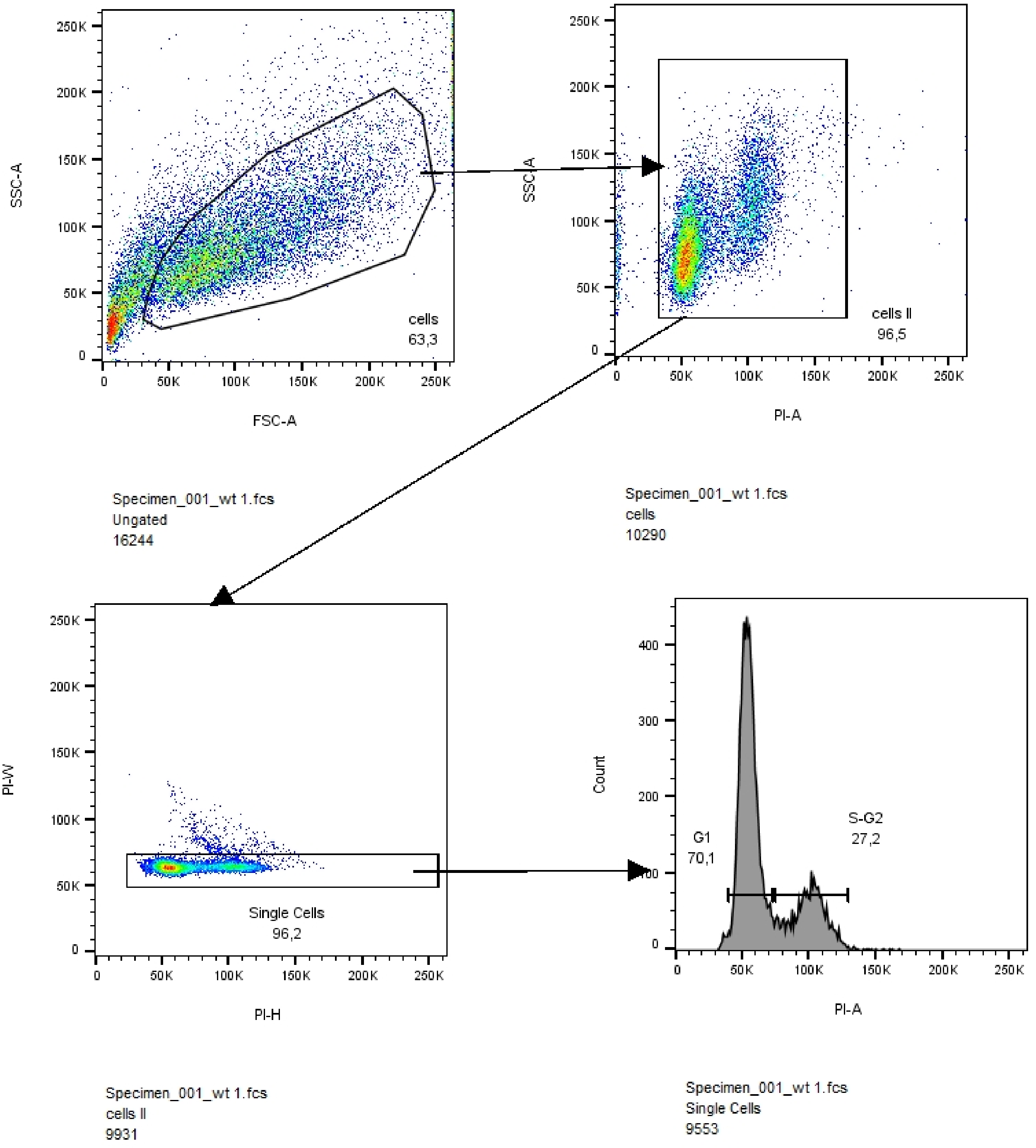

Journal of Cell Science • Supplementary information

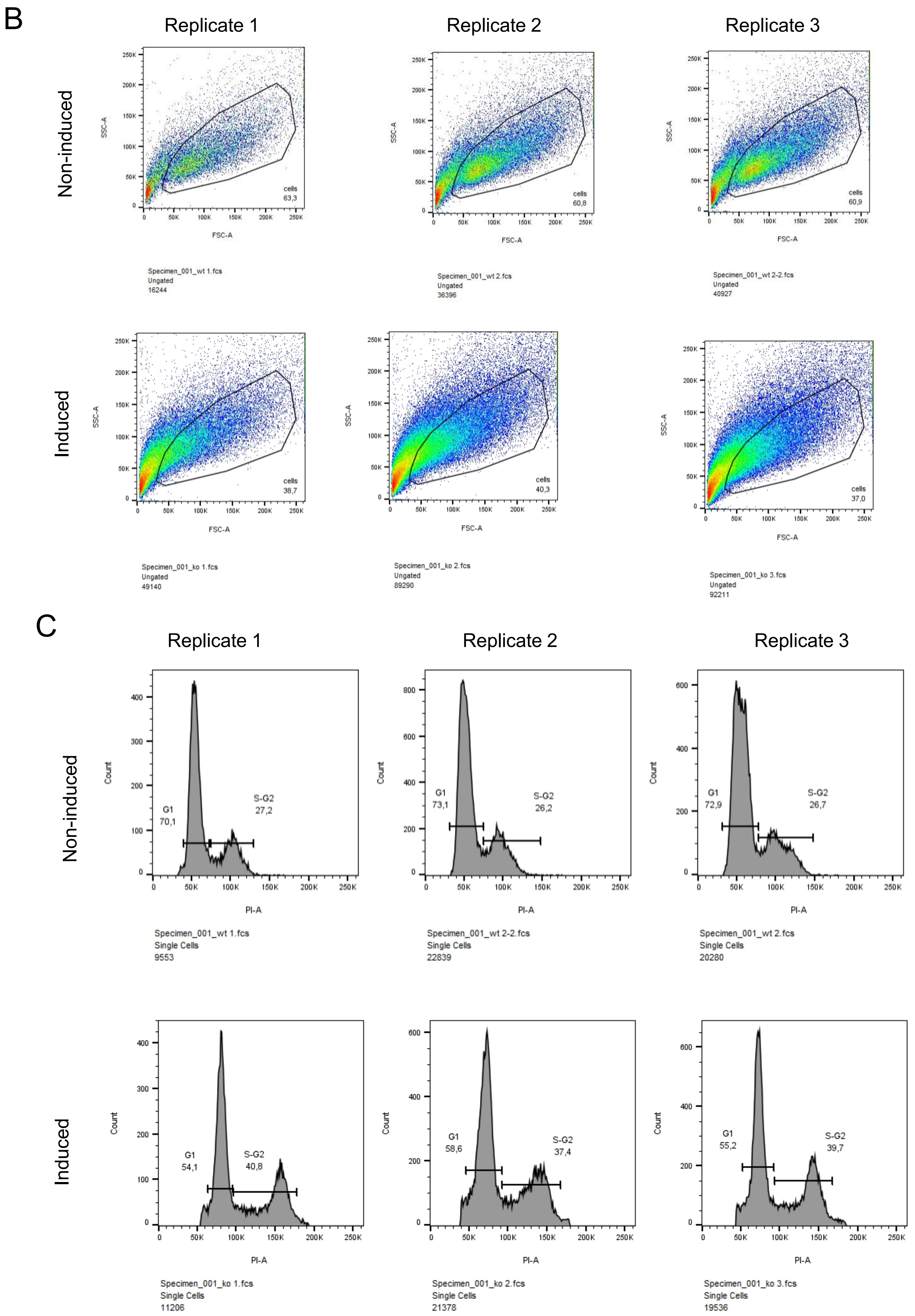

Fig. S4. FACS analysis of the DNA content. (A) Gating strategy for the analysis of DNA content. (B) Forward versus side scatter plots (FSC vs SSC) shows a different pattern between induced and non-induced parasites. (C) Flow cytometry DNA content distribution in the cell cycle analysis assay. Cells were stained with propridium iodine (PI) for 30 minutes prior analysis using FACS.

Journal of Cell Science • Supplementary information

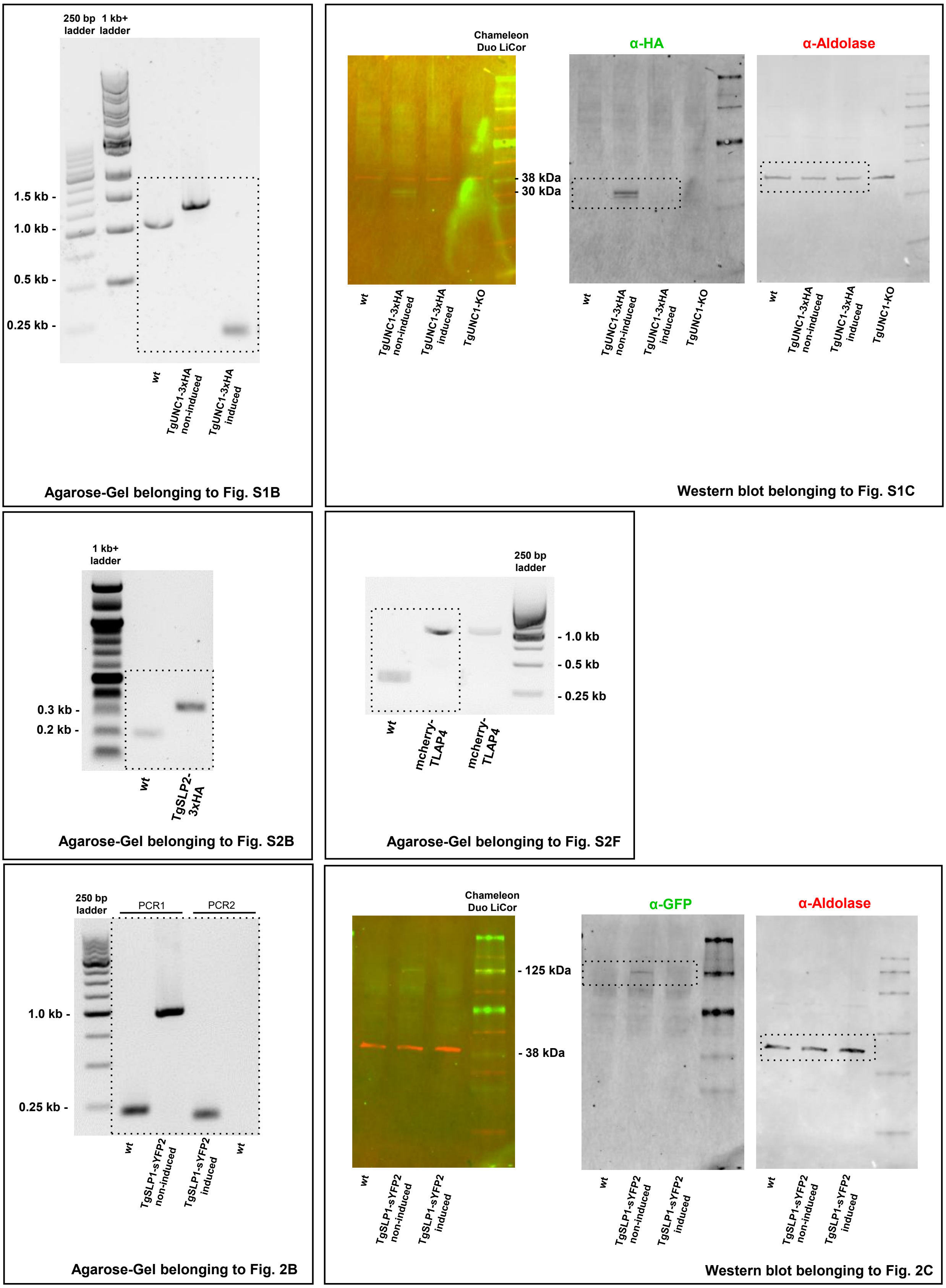

Fig. S5. Blot transparency. Uncropped western blots and agarose gels with molecular weight markers.

Journal of Cell Science • Supplementary information

Table S1. Sequences and binding positions within the genome of all sgRNAs generated in this study

Name	Binding position within genome	Sequence	PAM
TgUNC1_ sgRNA-Cterm-tag	2 bp upstream of STOP (revcom)	ACTTGCCAGCCGGTGATTCC	AGG
TgUNC1_sgRNA-Upstr-LoxP	3 bp upstream of START (revcom)	GAAGACAAAGATTGCAAAAC	CGG
TgSLP2_sgRNA-Cterm-tag	11 bp downstream STOP (revcom)	CTATTCGAACTCTAGTTAGC	TGG
TgSLP2_sgRNA-Upstr-LoxP(1)	8 bp downstream of START (revcom)	GCAGGCATCCCGCCCCCTCT	GGG
TgSLP2_sgRNA-Upstr-LoxP(2)	681 bp upstream of START, outside of 5UTR	CCGCTTCTTCCTTATCTTCT	CGG
TgSLP2_sgRNA-Upstr-LoxP(3)	1825 bp upstream of START, upstream of TGGT1_207110 (revcom)	GTTCAGCTGTGAATGTCAGC	TGG
TgSLP2_sgRNA-internal-LoxP	174 bp downstream of START	AGAGAGGGATCGAAAAGTGG	AGG
TgSLP2_sgRNA-Cterm-U1-silencing	1978 bp downstream of STOP	TTTCTATTGCCCGCTAGTAC	AGG
TgSLP1_sgRNA-Cterm-tag	14 bp downstream of STOP (revcom)	TTTCTGGGCATGCCTAGTTG	AGG
TgSLP1_sgRNA-Upstr-LoxP	8 bp upstream of START (revcom)	GTTTCTGGCTGCTCTGAGCA	CGG
TLAP4_sgRNA-Nterm-tag	6 bp upstream of START	TTTCCCGAAATTGCTCTGTT	TGG
CEP250_L1_sgRNA-Cterm-tag	22 bp upstream of STOP (revcom)	TGGCGATCCAGTCTCAACAG	TGG
Nuf2_sgRNA-Cterm-tag	8 bp upstream of STOP	CCGAGTAGAGCACCGATCT	TGG
Chromo1_sgRNA-Cterm-tag	37 bp downstream of STOP	CGACAGTGGACGAACCGGTC	AGG

Table S2. Oligonucleotides used in this study

Name	Sequence	Purpose
TgUNC1_sgRNA-Cterm-tag-fw	AAGTTGACTTGCCAGCCGGTGATTCCG	integration into Cas9YFP-sgRNA vector
TgUNC1_sgRNA-Cterm-tag-rv	AAAACGGAATCACCGGCTGGCAAGTCA	integration into Cas9YFP-sgRNA vector
TgUNC1_Cterm-tag-donor-fw	CGCCAGGTGTCCCTGAGGTGTCAGTGCACCTGGAGAGCCCCGC AGGGAAAGCTAAAATTGGAAGTGGAGG	PCR amplification of the repair template
TgUNC1_Cterm-tag-donor-rv	AAGCCACCGTTCCCTCAATTCGTACTACTCGTCACGGCTTCCTCA ACTCAATAACTTCGTATAATGTATGCTATACG	PCR amplification of the repair template
TgUNC1_sgRNA-Upstr-LoxP-fw	AAGTTGAAGACAAAGATTGCAAAACG	integration into Cas9YFP-sgRNA vector
TgUNC1_sgRNA-Upstr-LoxP-rv	AAAACGTTTTGCAATCTTTGTCTTCA	integration into Cas9YFP-sgRNA vector
TgUNC1_Upstr-LoxP-donor	CTCGCGTCCTTTCTTTTTCGCTTCCGGTTTTGCAAATAACTTCGTA TAGCATACATTATACGAAGTTATTCTTTGTCTTCACAATGCTGCC GACGTATCA	repair template
TgUNC1_5UTR-fw	CTTATTCGCCGTGGAGTTCTG	genotyping
TgUNC1_internal-fw	GCAGGTAATTGGTGTCCAAGAG	genotyping
TgUNC1_3UTR-rv	GTCTCATGTTTGCCTCCGTG	genotyping
TgUNC1_Upstr-LoxP-contr-rv	GAAGACAAAGAATAACTTCGTATAATGTATGC	genotyping
TgSLP2_sgRNA-Cterm-tag-fw	AAGTTGTATTCGAACTCTAGTTAGCG	integration into Cas9YFP-sgRNA vector
TgSLP2_sgRNA-Cterm-tag-rv	AAAACGCTAACTAGAGTTCGAATACA	integration into Cas9YFP-sgRNA vector
TgSLP2_Cterm-tag-donor-fw	AGCGAGGAGCCCCACGGGTGGAAGAGGCAGCTGGAATGCTCC CAGCAAACGCTAAAATTGGAAGTGGAGG	PCR amplification of the repair template
TgSLP2_Cterm-tag-donor-rv	AGGACCCGAAGCTGACTGTTCTCCGTTTCTGTTGCTCTATTCGAA CTCTAATAACTTCGTATAATGTATGCTATACG	PCR amplification, repair template
TgSLP2_internal-fw	ACCTGTGAAGGACGCAAGAG	genotyping
TgSLP2_3UTR-rv	CTCTCGGTTTCCTCGTTCTGTC	genotyping
TgSLP1_sgRNA-Cterm-tag-fw	AAGTTGTTTCTGGGCATGCCTAGTTGG	integration into Cas9YFP-sgRNA vector

Journal of Cell Science • Supplementary information

TgSLP1_sgRNA-Cterm-tag-rv	AAAACCAACTAGGCATGCCCAGAAACA	integration into Cas9YFP-sgRNA vector
TgSLP1_Cterm-tag-donor-fw	GCCTGAGAGTCCACGGCGAGAAGGCGGTGCTGAAGTCCACCAC CCTCAACGCTAAAATTGGAAGTGGAGG	PCR amplification of the repair template
TgSLP1_Cterm-tag-donor-rv	AGCATGTGCGACTGCTTTGCTTTCTTTGCCTACGTTTCTGGGCAT GCCTAATAACTTCGTATAATGTATGCTATACG	PCR amplification of the repair template
TgSLP1_sgRNA-Upstr-LoxP-fw	AAGTTGTTTCTGGCTGCTCTGAGCAG	integration into Cas9YFP-sgRNA vector
TgSLP1_sgRNA-Upstr-LoxP-rv	AAAACTGCTCAGAGCAGCCAGAAACA	integration into Cas9YFP-sgRNA vector
TgSLP1_Upstr-LoxP-donor	GCCGCTGCTTCTCCTTCGCCGTGCTCAGAGCAGCATAACTTCGTA TAGCATACATTATACGAAGTTATCAGAAACATCCTGCGATGGAC TCCTTCGAGCG	repair template
TgSLP1_5UTR-fw	CAGCGGGCTTCTGTATTTGC	genotyping
TgSLP1_internal-fw	CTGAAGGAGAAGCCGGTACG	genotyping
TgSLP1_3UTR-rv	GCAGTTGGGCATTCCATTTCG	genotyping
TgSLP1_Upstr-LoxP-contr-rv	GATGTTTCTGATAACTTCGTATAATGTATGC	genotyping
TLAP4_sgRNA-Nterm-tag-fw	AAGTTGTTTCCCGAAATTGCTCTGTTG	integration into Cas9YFP-sgRNA vector
TLAP4_sgRNA-Nterm-tag-rv	AAAACAACAGAGCAATTTCGGGAAACA	integration into Cas9YFP-sgRNA vector
TLAP4_Nterm-tag-donor-fw	CGGGACTTCTCCCTGTGTCTCTCGCGAAAAAACCCTGGGTTCCA AACAGAAATTTTTTATGGTGAGCAAGGGCG	PCR amplification of the repair template
TLAP4_Nterm-tag-donor-rv	CATTTTGCAGAGATGTCCACTGCGTTCCATAAAAAAATTTCCCGAA ATTGCCCTTGTACAGCTCGTCCATGC	PCR amplification of the repair template
TLAP4_5UTR-fw	GGTTTCAACGCTTCTTCTTCG	genotyping
TLAP4_internal-rv	CTACATCGAAGAATGCCACCT	genotyping
CEP250_L1_sgRNA-Cterm-tag-fw	AAGTTGTGGCGATCCAGTCTCAACAGG	integration into Cas9YFP-sgRNA vector
CEP250_L1_sgRNA-Cterm-tag-rv	AAAACCTGTTGAGACTGGATCGCCACA	integration into Cas9YFP-sgRNA vector
CEP250_L1_sgRNA-Cterm-tag-donor-fw	CTTCGACAAGGATCAGCACAGGAAGGGGAAAGTCGTCGCTTTC GGGGCGAGCTAAAATTGGAAGTGGAGG	PCR amplification of the repair template
CEP250_L1_sgRNA-Cterm-tag-donor-rv	TGCTCGTCCATAAACATCGCTACTGCAGAGGCTTGGCGATCCAG TCTCAAATAACTTCGTATAATGTATGCTATACG	PCR amplification of the repair template
CEP250_L1_5UTR-fw	CACCTGTCCGCTTTCAATTC	genotyping
CEP250_L1_internal-rv	CAACACTCTACGAACCTGTC	genotyping
Nuf2_sgRNA-Cterm-tag-fw	AAGTTGCCGAGTAGAGCACCGATCTG	integration into Cas9YFP-sgRNA vector
Nuf2_L1_sgRNA-Cterm-tag-rv	AAAACAGATCGGTGCTCTACTCGGCA	integration into Cas9YFP-sgRNA vector
Nuf2_sgRNA-Cterm-tag-donor-fw	CAAGGGAGCCCAGAGAAGACGGCGACTTTCCAATGTATAGTCA CGCCGAGGCTAAAATTGGAAGTGGAGG	PCR amplification of the repair template
Nuf2_sgRNA-Cterm-tag-donor-rv	GCCAAAGTTCTCCGAGTGTCCGTACACCGGAAACTTTCTCCATG CCAAGAATAACTTCGTATAATGTATGCTATACG	PCR amplification of the repair template
Nuf2_5UTR-fw	AGCGAGAACGAGAATCCGAC	genotyping
Nuf2_internal-rv	CGAGTGTCCGTACACCGGAA	genotyping
Chromo1_sgRNA-Cterm-tag-fw	AAGTTGCGACAGTGGACGAACCGGTCG	integration into Cas9YFP-sgRNA vector
Chromo1_sgRNA-Cterm-tag-rv	AAAACGACCGGTTCGTCCACTGTCGCA	integration into Cas9YFP-sgRNA vector
Chromo1_sgRNA-Cterm-tag-donor-fw	CCGTCTGCGGGAGGTTCCAACGCTCTCCAGTGTTCTCTTGTGTC GCTGGCGCTAAAATTGGAAGTGGAGG	PCR amplification of the repair template
Chromo1_sgRNA-Cterm-tag-donor-rv	CCAGTCTCGTGGAAGCGGTGCAACGCCTCTGGGATTCCGTTCGG CCTGACATAACTTCGTATAATGTATGCTATACG	PCR amplification of the repair template
Chromo1_5UTR-fw	CGTTCGTTTCAGATGAGTCC	genotyping
Chromo1_internal-rv	GGTATAGAGACAGCGCGTTG	genotyping

Journal of Cell Science • Supplementary information

Table S3. Antibodies and their origin used in this study

**Primary Antibodies**	**Species**	**Dilution**	**Reference**
Aldolase	Rabbit	1:2000	Sibley, L.D.
Centrin1	Mouse	1:1000	Sigma 04-1624
GAP45	Rabbit	1:5000	Soldati, D.
GFP	Mouse	1:1000	Roche 11814460001
GFP	Rabbit	1:1000	Abcam #ab290
HA	Rat	1:1000	Roche 11867423001
IMC1	Mouse	1:1000	Ward, G.
MIC2	Mouse	1:500	Carruthers, V.
MIC8	Rabbit	1:500	Soldati, D.
ROP2,4	Mouse	1:500	Dubremetz, J.F.
**Secondary Antibodies**	**Species**	**Dilution**	**Reference**
AlexaFluor 350 goat-anti-rabbit	Goat	1:5000	Invitrogen A-11046
AlexaFluor 488 goat-anti-mouse	Goat	1:5000	Invitrogen A-11001
AlexaFluor 488 goat anti-rabbit	Goat	1:3000	Invitrogen A-11008
AlexaFluor 546 goat-anti-mouse	Goat	1:3000	Invitrogen A-11030
AlexaFluor 594 goat-anti-mouse	Goat	1:5000	Invitrogen A-11005
AlexaFluor 594 goat-anti-rabbit	Goat	1:5000	Invitrogen A-11012
AlexaFluor 488 goat-anti-rat	Goat	1:5000	Invitrogen A-11006
AlexaFluor 594 goat-anti-rat	Goat	1:5000	Invitrogen A-11007
AlexaFluor 647 chicken anti-rat	Chicken	1:3000	Invitrogen A-21472
IRDye680RD goat-anti-rabbit	Goat	1:1000	Li-Cor 026-68071
IDRye800CW goat-anti-rat	Goat	1:1000	Li-Cor 926-32219
IDRye800CW goat-anti-mouse	Goat	1:1000	Li-Cor 926-32210

Journal of Cell Science • Supplementary information

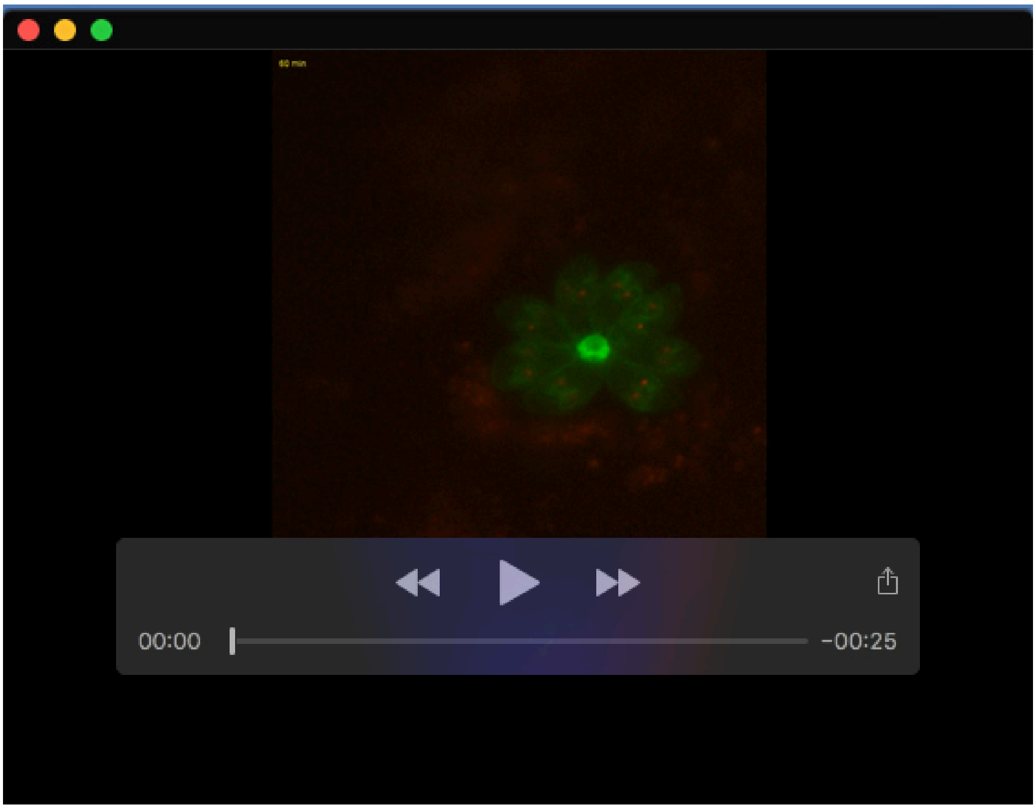

Movie 1. Time-lapse analysis of TgSLP1 and F-actin localisation during parasite division. TgSLP1 was C-terminally tagged with mCherry in a parasite line expressing CbEmerald to visualise F-actin.

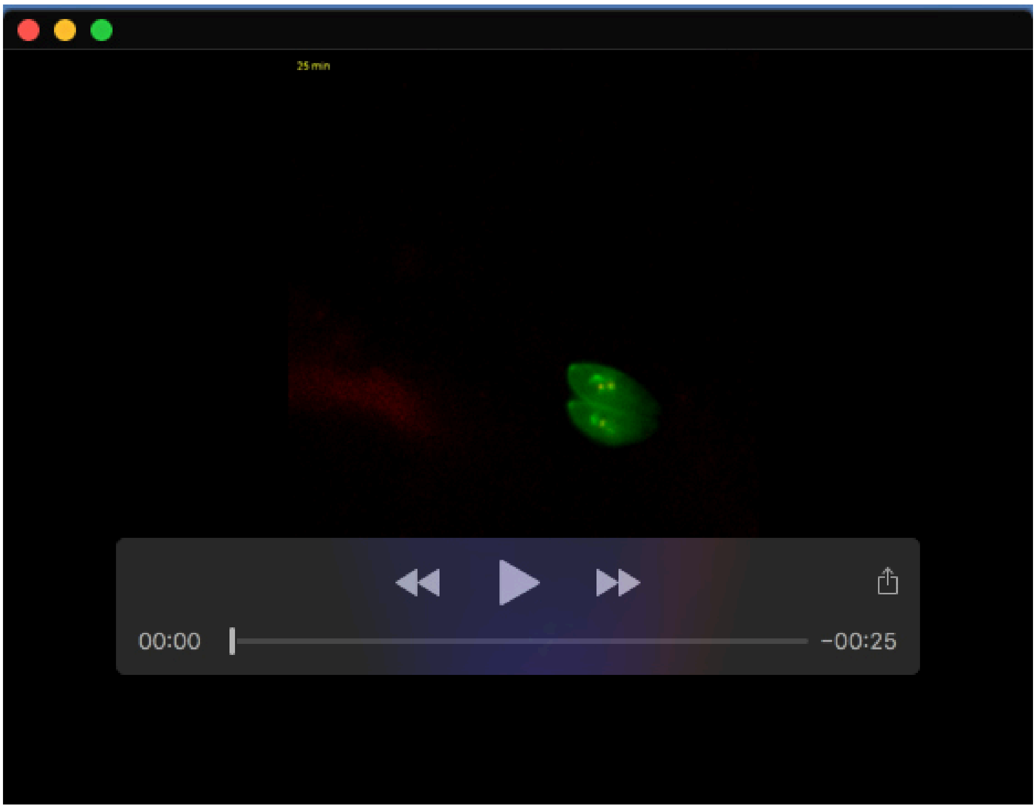

Movie 2. Time-lapse analysis of TgSLP1 and α-tubulin localisation during parasite division. TgSLP1 was C-terminally tagged with sYFP2 in a parasite line transiently expressing α-tubulin tagged with mCherry to visualise microtubular structures.

Journal of Cell Science • Supplementary information
