# Supplementary material for: The SUN-like protein TgSLP1 is essential for nuclear division in the apicomplexan parasite Toxoplasma gondii
Source: J Cell Sci. 2023 Oct 30;136(21):jcs260337. doi: 10.1242/jcs.260337 (PMC10629696; doi:10.1242/jcs.260337)
Supplement: Supplementary file 1 [file joces-136-260337_review_history.pdf]

Journal of Cell Science

The SUN-like protein TgSLP1 is essential for nuclear division in the apicomplexan parasite *Toxoplasma gondii*

Mirjam Wagner, Yuan Song, Elena Jiménez-Ruiz, Sonja Härtle and Markus Meissner

DOI: 10.1242/jcs.260337

Editor: David Glover

Review timeline
Original submission: 14 June 2022
Editorial decision: 1 August 2022
First revision received: 2 August 2023
Accepted: 22 September 2023

Original submission

First Decision Letter

MS ID#: JOCES/2022/260337

MS TITLE: The SUN-like protein TgSLP1 is essential for nuclear division in the apicomplexan parasite Toxoplasma gondii

AUTHORS: Mirjam Wagner, Elena Jimenez-Ruiz, and Markus Meissner
ARTICLE TYPE: Research Article

We have now reached a decision on the above manuscript.

To see the reviewers' reports and a copy of this decision letter, please go to: https://submit-jcs.biologists.org and click on the 'Manuscripts with Decisions' queue in the Author Area. (Corresponding author only has access to reviews.)

As you will see, the reviewers raise a number of substantial criticisms that prevent me from accepting the paper at this stage. They suggest, however, that a revised version might prove acceptable, if you can address their concerns. If you think that you can deal satisfactorily with the criticisms on revision, I would be pleased to see a revised manuscript. We would then return it to the reviewers.

Please ensure that you clearly highlight all changes made in the revised manuscript. Please avoid using 'Tracked changes' in Word files as these are lost in PDF conversion.

Reviewer 1

Advance summary and potential significance to field

Characterizing the nuclear envelope LINC complex in T. gondii is the goal of this manuscript, in particular the putative SUN and KASH orthologs. The KASH candidates are discussed in the discussion, but no data is shown (all non-shown data described indicate roles outside the nucleus), whereas SUN candidates SLP1 and SLP2 as well as the UNC1 ortholog are experimentally interrogated here. TgUNC1 resides in the Golgi, SLP2 is present in cytoplasmic and vacuolar focus, an SLP1 is the only one present in the nucleus. Further experimental dissection of SLP1 shows it is only present in the nucleus when the spindle microtubules are present (although that is not super-

© 2023. Published by The Company of Biologists under the terms of the Creative Commons Attribution License (https://creativecommons.org/licenses/by/4.0/).

firm in the data presented), and seems to be associated with the nuclear envelope around the spindle. Depletion of SLP1 results in nuclear division and partitioning defects, and somewhat surprisingly also a centrosome integrity (or partitioning?) defect. This latter is somewhat surprising as the apicoplast largely partitions normally, which is believed to partition in association with the centrosome (not explored exhaustively in the data presented though), and even daughter budding is largely coordinated by the centrosome, which is largely normal as well. It is therefore possible centrosome, or at least the Centrin1 reporter used, loose integrity as a secondary defect, and is not a primary defect. The use of the diCre system to deplete SLP1 has however slow kinetics, and therefore it is not easy to differentiate primary from secondary defects. At least the inner-core of the centrosome, which coordinates the nuclear cycle should be explored in this context. Finally, the schematics in Fig 7 contain some unclarities and speculations that needs attention. Overall, this is an interesting study showing that the typical LINC complex components are have unusual and deviating roles in T. gondii.

Comments for the author

Specific points

1. Long rapamycin induction times (24 hrs in some cases, but most cases 48 hrs) make it hard to differentiate primary from secondary defects in light of a 6-7 hr cell division cycle, which is inherent to the relatively slow kinetγics of the diCre system; fast kinetics of mAID might be more insightful.
2. Fig 6D: centrin 1 and apicoplast association is lost, whereas in Fig 6B almost all daughter parasites have an apicoplast. That raises the question whether the apicoplast is indeed tethered to the centrosome to secure its partitioning or not (the going model); alternative interpretations are that either the centrosome falls apart after completion of this round of cell division, or that just centrin 1 becomes fragmented but the rest of the centrosome stays intact. The phenotype of losing the (outer core) centrosome somewhat contrasts with the CEP250L1 depletion (inner core) recently presented by the Francia lab at the Toxomeeting (not published as yet), which did not seem to impact the outer core (but karyokinesis was blocked, like seen here for SLP1). It is highly recommended to visualize the inner-core of the centrosome (and/or other centrosome markers) in the SLP1-KO mutant to shed further light on how SLP1, which acts in the nuclear cycle, interfaces/impacts with the centrosome inner-core, which coordinates the nuclear/mitotic cycle.
3. Related to the above: where is centrocone-MORN1 in the SLP1-KO? This could potentially still be associated with the nucleus/centrocone but if not, it could indicate that the spindle does not form and/or does its job in separating the chromosomes clustered at their kinetochores.
4. Fig 7A. It has been demonstrated that here is no mitotic spindle during G1 phase (PMID: 26466679), which is incorrectly represented in the figure.
5. Fig 7B. The depicted model does not take into account the role of kinetochores in anchoring the nucleus to the centrosome during mitosis as previously shown (PMID: 24015880); moreover, nucleus anchoring to the centrosome does not require microtubules during interphase. Since the KASH candidate is missing, this model is highly speculative to the point where it is misleading. I strongly suggest to remove this model.

Minor points.

6. Vps31 geneID is not included in the manuscript; this appears to be the first report, although it is annotated in ToxoDB, referring to PMID 26175406, but there is no experimental validation in that manuscript.
7. Fig 5/6. Does SLP1 localization to the spindle require microtubules? What if oryzalin was added during HU treatment for example?
8. Fig 5B. It is highly recommended to include some of the HU treated IFA samples, as this is not a standard/established approach in T. gondii and reportedly kills the parasites very rapidly (i.e. artefacts might be likely or at least should be excluded).
9. Line 367 M&M; cell cycle arresting drugs. It is not clear whether parasites were exposed for 24, 6 or 30 hrs? Looks like they were first grown without drugs for 24 hrs, but that is not clearly stated.

© 2023. Published by The Company of Biologists under the terms of the Creative Commons Attribution License (https://creativecommons.org/licenses/by/4.0/).

Reviewer 2

Advance summary and potential significance to field

This is a manuscript from Mirjam Wagner et al. describing the characterization of three UNC family proteins in the apicomplexan parasite Toxoplasma gondii. The authors describe the localization of these proteins. They also produce inducible Knock-out mutants for two of these genes and investigate their phenotypes. T. gondii presents a peculiar cell cycle and nuclear division but emerges as a simplified model to study these processes in eukaryotes. Interestingly, the mechanisms underlying the segregation of the centromeres at the nuclear periphery at all phase of the cell cycle is not understood. Therefore, investigating the UNC family proteins is timely and may provide new insights into cell cycle regulation in this organism and other eukaryotes. This study is nicely performed and provides substantial evidence for the localization of the proteins but unfortunately, phenotype description is only partial and lacks important pieces of data. An in-depth study of the TgSLP1 inducible KO phenotype is required.

Comments for the author

Major concerns

- The authors did not evaluate the effect of rapamycin treatment on the WT parasites. It is important to produce data showing that Rapamycin is not the only cause of the phenotypes observed
- While the cell cycle regulation of TgSLP1 is nicely described, its localization is still uncertain. This is mainly due to the use of overexpressed (αTubulin and MORN1) or oddly chosen (TLPA4 and VPS31) markers. Yet, the authors mention that “TgSLP1 localises at the mitotic spindle” (lines 243-244). As such, this statement is not supported by data. There is a large panel of well-characterized markers that can be used to precisely characterize TgSLP1 localization. TgSLP1 should be localized together with an inner core centrosome (Cep250), kinetochore (Ndc80 or Nuf2), spindle microtubules (EB1), and centromeres (CenH3 or Chromo1).
- The author should provide data on the dynamic of the Cre/lox KO inducible system. How many parasites have the TgSLP1 locus excised after 24 hours of rapamycin treatment?
- The phenotypes of the inducible KO of TgSLP1 are only partially characterized. In Figure 6B, there is no statistical analysis of the phenotypes. The centrin1 phenotype (Figure 6D) is not quantified and the only picture provided is hard to interpret.
- To better understand the role of TgSLP1, the authors need to investigate the fate of the inner core centrosome, kinetochore, spindle microtubules, and centromeres in presence of rapamycin.

Minor:

- Plaque assays could be quantified to verify that there is no effect of the insertion of the loxP sites in the TgUNC1 locus.
- Electron microscopy analysis of the mutant in presence of rapamycin would permit the reader to draw a sharper picture of the phenotypes.
- FACS analysis of the DNA content is a standard to analyze the cell cycle defects of T. gondii mutants. The authors should investigate the mutant phenotype using this tool.

Reviewer 3

Advance summary and potential significance to field

In this study, Wagner et al described three homologues of UNC family proteins in the apicomplexan parasite T. gondii. The authors showed that the UNC-50 protein TgUNC1 localised to the Golgi and appeared to be not essential for the parasite and the SUN domain protein TgSLP2 showed a signal throughout the parasite and its biological roles have not been found. In constrast, the second SUN domain protein named TgSLP1 was expressed in a cell cycle-dependent manner and colocalised with the mitotic spindle and the centrocone. The conditional knockout of TgSLP1 leads to failure of nuclear division and loss of centrosome integrity.

© 2023. Published by The Company of Biologists under the terms of the Creative Commons Attribution License (https://creativecommons.org/licenses/by/4.0/).

The work is interesting and the data are quite convincing. However, this work requires some modifications and additionnal experiments that will improve the quality and impact of the paper.

Comments for the author

Here are my major concerns:
1- It is not clear why the two first proteins TgUNC1 and TgSLP2 whose characterization add nothing to the functionally importance of TgSLP1 should be shown as main figures. No speficic and important functions are discovered for these two first proteins despite many efforts to characterize them by confocal microsocpy and gene knockout. Therefore, I will suggest to show the data of TgUNC1 and TgSLP2 described in figures 1, 2 and 3 (out of 7 main figures) as supplemenatry figures. The authors could quickly and briefly indicated in the text and also in the discussion section that they do not discover any important functions for TgUNC1 and TgSPL2, therefore they focus their attention and work more on TgSLP1 that is an essential protein for parasite's nuclear division.
2- The link between TgSLP1 and VPS31 is not clearly explained or no solid experimental evidence has been provided to justify why VPS31 should be studied and presented herein. In addition, the authors do not mention this protein later on in the discussion paragraph.
3- I am wondering why electron microscopy (EM) has not been done to see the ultrastructural morphology and modifications that occured in the knock out TgSLP1 mutant. Only confocal microscopy and antibodies have been used and EM will be more informative to see the ultractural sections of mutant, for examples, the parasite's nucleus leaving the cytoplasm and being located between the plasmalemma and inner membrane complex (IMC) as stated by the authors, and even new phenotypes.
4- I am quite surprised that the authors have only relied on « educational guess » by looking at putative homologues in genome database of T. gondii instead of using the epitope tagged TgSLP1 transgenic parasites to identify TgSLP1 partners by cross-linking experiments following by mass spectrometry analyses to discover potential KASH-like proteins that seems to be absolutely required for the proper functions of TgSLP1. These kind of experiments will seriously improve the impact of this work.

Minor concerns:
1- Fig S2 depicted diagrams of the strategies used to invalide these genes and I am not sure if this is neccessary to be shown.
2- Is there any guess that the classical ATc-inducible knock out system may work when LoxP, U1SnRNP-meduiated silencing and auxin-inducible degron system failed for TgSLP2 ?
3- The GRA1 confocal micrsocopy shown in Fig S1 in not convincing.

Normally, the GRA1 signal usually decorates very nicely the PVM and it was not the case here. Otherwise, my opinion is that this manuscript is interesting but requires a revision in its presentation and additionnal experiments before publication in JCS.

First revision

Author response to reviewers' comments

Reviewer 1 Advance Summary and Potential Significance to Field:

Characterizing the nuclear envelope LINC complex in *T. gondii* is the goal of this manuscript, in particular the putative SUN and KASH orthologs. The KASH candidates are discussed in the discussion, but no data is shown (all non-shown data described indicate roles outside the nucleus), whereas SUN candidates SLP1 and SLP2 as well as the UNC1 ortholog are experimentally interrogated here. TgUNC1 resides in the Golgi, SLP2 is present in cytoplasmic and vacuolar focus, an SLP1 is the only one present in the nucleus. Further experimental dissection of SLP1 shows it is only present in the nucleus when the spindle microtubules are present (although that is not super-

© 2023. Published by The Company of Biologists under the terms of the Creative Commons Attribution License (https://creativecommons.org/licenses/by/4.0/).

firm in the data presented), and seems to be associated with the nuclear envelope around the spindle.

Depletion of SLP1 results in nuclear division and partitioning defects, and somewhat surprisingly also a centrosome integrity (or partitioning?) defect. This latter is somewhat surprising as the apicoplast largely partitions normally, which is believed to partition in association with the centrosome (not explored exhaustively in the data presented though), and even daughter budding is largely coordinated by the centrosome, which is largely normal as well. It is therefore possible centrosome, or at least the Centrin1 reporter used, loose integrity as a secondary defect, and is not a primary defect. The use of the diCre system to deplete SLP1 has however slow kinetics, and therefore it is not easy to differentiate primary from secondary defects. At least the inner-core of the centrosome, which coordinates the nuclear cycle should be explored in this context. Finally, the schematics in Fig 7 contain some unclarities and speculations that needs attention.

Overall, this is an interesting study showing that the typical LINC complex components are have unusual and deviating roles in *T. gondii*.

We thank the reviewer for his time to critically assess our study and the positive criticism that helped to improve this manuscript. Please find our detailed response below.

Reviewer 1 Comments for the Author:
Specific points
1. Long rapamycin induction times (24 hrs in some cases, but most cases 48 hrs) make it hard to differentiate primary from secondary defects in light of a 6-7 hr cell division cycle, which is inherent to the relatively slow kinetics of the diCre system; fast kinetics of mAID might be more insightful.

The reviewer rightfully mentions that faster regulation systems can be of advantage to differentiate direct from indirect phenotypes, especially when it comes to stable proteins with a long half-life. Given that TgSLP1 is cell cycle dependent and undetectable in parasites entering G1-phase, as shown by our detailed analysis of the cell cycle dependent expression, we argue that upon excision of the gene, no protein is formed *de novo* and old proteins will be degraded in G1. Therefore, based on our experience with similar proteins the use of the AID system would be only of marginal advantage.

2. Fig 6D: centrin 1 and apicoplast association is lost, whereas in Fig 6B almost all daughter parasites have an apicoplast. That raises the question whether the apicoplast is indeed tethered to the centrosome to secure its partitioning or not (the going model); alternative interpretations are that either the centrosome falls apart after completion of this round of cell division, or that just centrin 1 becomes fragmented but the rest of the centrosome stays intact. The phenotype of losing the (outer core) centrosome somewhat contrasts with the CEP250L1 depletion (inner core) recently presented by the Francia lab at the Toxomeeting (not published as yet), which did not seem to impact the outer core (but karyokinesis was blocked, like seen here for SLP1). It is highly recommended to visualize the inner-core of the centrosome (and/or other centrosome markers) in the SLP1-KO mutant to shed further light on how SLP1, which acts in the nuclear cycle, interfaces/impacts with the centrosome inner-core, which coordinates the nuclear/mitotic cycle.

We thank the reviewer for this suggestion. Previous work by Francia et al. in 2012 demonstrated that *Toxoplasma* division depends on the SFA fibre that provides robust spatial and temporal organisation of division. In absence of TgSLP1 the apicoplast duplication and division can still occur but is uncoupled from karyokinesis. For our revision, we performed multiple transfections and generated double-tagged lines with TgSLP1 and TgCEP250_L1 (marker for the inner core centrosome), Chromo1 (centromeres) and Nuf2 (kinetochore). These data are now presented in Figure 5.

Briefly, we demonstrate that TgSLP1 divides after the outer and inner core of the centrosome, before the centromeres but at the same time as the kinetochore, suggesting that TgSLP1 is involved in kinetochore clustering as observed in other eukaryotes.

© 2023. Published by The Company of Biologists under the terms of the Creative Commons Attribution License (https://creativecommons.org/licenses/by/4.0/).

Furthermore, we analysed the effect of TgSLP1 depletion on these markers and it appears that centrin1 and Cep250_L1 remain in close association but Nuf2 and Chromo1 show aberrant localisation in regard to centrin1, suggesting that TgSLP1 connects the kinetochore/ centromeres with the centrosome. These results are now shown in Figure 5.

3. Related to the above: where is centrocone-MORN1 in the SLP1-KO? This could potentially still be associated with the nucleus/centrocone but if not, it could indicate that the spindle does not form and/or does its job in separating the chromosomes clustered at their kinetochores.

This is a very interesting question that we wished to address but failed since transient transfection of parasites expressing fluorescent MORN1 often resulted in overexpression phenotypes, making it difficult to distinguish between the effect of the TgSLP1-KO and the overexpression phenotype of MORN1. Unfortunately, despite several attempts, we have not been able to generate a stable, double-tagged cell line of TgSLP1 and MORN1.

4. Fig 7A. It has been demonstrated that here is no mitotic spindle during G1 phase (PMID: 26466679), which is incorrectly represented in the figure.

We thank the reviewer for pointing this out. We have revised the whole Figure which is now presented in Figure 5.

5. Fig 7B. The depicted model does not take into account the role of kinetochores in anchoring the nucleus to the centrosome during mitosis as previously shown (PMID: 24015880); moreover, nucleus anchoring to the centrosome does not require microtubules during interphase. Since the KASH candidate is missing, this model is highly speculative to the point where it is misleading. I strongly suggest to remove this model.

We now performed detailed analysis of the centrosome, kinetochore and centromeres using STED microscopy (Fig.5) and changed the model accordingly to better reflect the data.

Minor points.

6. Vps31 geneID is not included in the manuscript; this appears to be the first report, although it is annotated in ToxoDB, referring to PMID 26175406, but there is no experimental validation in that manuscript.

We initially thought to present this subunit of the ESCRTIII complex as an additional, interesting set of data. However, during the revision of this manuscript, we continued our analysis of ESCRTIII functions, which will now be the topic of an independent, future study. Therefore, we removed the VPS31 data.

7. Fig 5/6. Does SLP1 localization to the spindle require microtubules? What if oryzalin was added during HU treatment for example?

Oryzalin treatment results in depolymerisation of all microtubules. The changes are serious for almost all markers, including TgSLP1. However, we now show STED and widefield images of these treatments in Figure 3E and supplementary Figure S3C respectively. TgSLP1 is still present near the centrosome (anti-centrin1 marker) even in the absence of microtubules although its duplication is prevented by the lack of spindle. Presence of HU and oryzalin did not alter this phenomenon. Indeed, HU treatment increased the intensity and percentage of parasites with TgSLP1 signal as also shown in Figure 3B and supplementary Figure S3B.

8. Fig 5B. It is highly recommended to include some of the HU treated IFA samples, as this is not a standard/established approach in *T. gondii* and reportedly kills the parasites very rapidly (i.e. artefacts might be likely or at least should be excluded).

As suggested, we included IFA images of the HU/ PDTC treated samples to exclude artefacts in supplementary Figure S3B.

9. Line 367 M&M; cell cycle arresting drugs. It is not clear whether parasites were exposed for 24,

© 2023. Published by The Company of Biologists under the terms of the Creative Commons Attribution License (https://creativecommons.org/licenses/by/4.0/).

6 or 30 hrs? Looks like they were first grown without drugs for 24 hrs, but that is not clearly stated.

The parasites were grown without drugs for 24 hours and after exposing the drugs for 6 hours, the samples were directly fixed and processed. We have specified this in the manuscript (see point Materials and Methods; *cell cycle arresting and microtubule depolymerisation drugs*).

Reviewer 2 Advance Summary and Potential Significance to Field:
This is a manuscript from Mirjam Wagner et al. describing the characterization of three UNC family proteins in the apicomplexan parasite *Toxoplasma gondii*. The authors describe the localization of these proteins. They also produce inducible Knock-out mutants for two of these genes and investigate their phenotypes. *T. gondii* presents a peculiar cell cycle and nuclear division but emerges as a simplified model to study these processes in eukaryotes. Interestingly, the mechanisms underlying the segregation of the centromeres at the nuclear periphery at all phase of the cell cycle is not understood. Therefore, investigating the UNC family proteins is timely and may provide new insights into cell cycle regulation in this organism and other eukaryotes. This study is nicely performed and provides substantial evidence for the localization of the proteins but unfortunately, phenotype description is only partial and lacks important pieces of data. An in-depth study of the TgSLP1 inducible KO phenotype is required.

We thank the reviewer for the time to critically assess our study and the positive criticism that helped to improve our manuscript. Please find our detailed response below.

Reviewer 2 Comments for the Author:
Major concerns
1. The authors did not evaluate the effect of rapamycin treatment on the WT parasites. It is important to produce data showing that Rapamycin is not the only cause of the phenotypes observed

We thank the reviewer for this comment. These control experiments have all been performed within this study and in several previous studies, since the establishment of the DiCre-System (Andenmatten et al., 2013).

Rapamycin is also used for the recently established splitCas9-system (Li et al., 2022). While rapamycin has effects at high concentrations (> 2 µM), at the concentrations used (50-100 nM), no effects can be detected in host cells or wildtype parasites, making it one of the "gold standard" inducers in *Toxoplasma* and other eukaryotes.

We also would like to refer the reviewer to Fig. 4A and supplementary Fig. S1D, where plaque assay on wildtype parasites is shown in presence and absence of rapamycin, confirming above mentioned publications that rapamycin has no effect at the concentrations used. We also mention this in the manuscript now (lines 222-223).

2. While the cell cycle regulation of TgSLP1 is nicely described, its localization is still uncertain. This is mainly due to the use of overexpressed (αTubulin and MORN1) or oddly chosen (TLPA4 and VPS31) markers. Yet, the authors mention that "TgSLP1 localises at the mitotic spindle" (lines 243-244). As such, this statement is not supported by data. There is a large panel of well-characterized markers that can be used to precisely characterize TgSLP1 localization. TgSLP1 should be localized together with an inner core centrosome (Cep250), kinetochore (Ndc80 or Nuf2), spindle microtubules (EB1), and centromeres (CenH3 or Chromo1).

We thank the reviewer for these suggestions. For our revision we performed multiple transfections and generated double-tagged lines with TgSLP1 and TgCEP250_L1 (inner core centrosome), Chromo1 (centromeres) and Nuf2 (kinetochore). These data are now presented in Figure 5A. Please see also our reply to reviewer 1, question 2. In the light of this new data, we can now confidently say that TgSLP1 localises near the kinetochores, and it is important for their division and subsequently for the division of the centromeres.
Furthermore, we suggest that TgSLP1 links the centromeres and kinetochore to the centrosome.

3. The author should provide data on the dynamic of the Cre/lox KO inducible system. How many

© 2023. Published by The Company of Biologists under the terms of the Creative Commons Attribution License (https://creativecommons.org/licenses/by/4.0/).

parasites have the TgSLP1 locus excised after 24 hours of rapamycin treatment

After 24 hours of rapamycin treatment, we still saw some residual protein levels in immunofluorescence assays, probably reflecting undivided parasites (see supplementary Fig. S3D). Consequently, we decided to perform all assays after 48 hours of rapamycin treatment.

4. The phenotypes of the inducible KO of TgSLP1 are only partially characterized. In Figure 6B, there is no statistical analysis of the phenotypes. The centrin1 phenotype (Figure 6D) is not quantified and the only picture provided is hard to interpret.

We performed additional experiments and statistical analysis has been added now to the graphs (Fig. 3B, 4C, supplementary Fig. S3A). In the case of centrin1 the quantification was based on normal or abnormal localisation/ morphology of the marker since every vacuole presented a slightly different appearance (Fig. 4F).

5. To better understand the role of TgSLP1, the authors need to investigate the fate of the inner core centrosome, kinetochore, spindle microtubules, and centromeres in presence of rapamycin.

These experiments have now been performed, see Figure 5C and F. In the light of the new data, TgSLP1 seems to be responsible for kinetochore clustering and centromere association to the centrosome during cell division.

Minor:
6. Plaque assays could be quantified to verify that there is no effect of the insertion of the loxP sites in the TgUNC1 locus.

We demonstrated that TgUNC1 localised to the Golgi and can be removed without any apparent effects. Surprisingly (and against our prediction), we were able to isolate a null mutant for *unc1* that grows normally, at least *in vitro*. Although we cannot exclude a role of this protein in overall growth of the parasite, as mentioned by the reviewer, we did not perform further analysis of this mutant, such as competition assays. Since the null mutant did not demonstrate any morphological defects (see supplementary Fig. S1) and its “wrong” localisation at the Golgi, we concluded that this protein is not part of LINC.

We decided to now show the data in the supplements, as additional information for the reader (see also our response to reviewer 3, question 1).

7. Electron microscopy analysis of the mutant in presence of rapamycin would permit the reader to draw a sharper picture of the phenotypes.

We appreciate the reviewer’s suggestion. Based on our experience with EM imaging, we have determined that it would not provide us additional information in this particular case. Instead, we have chosen to utilise STED super-resolution microscopy to examine TgSLP1 and markers associated with the centrosome, kinetochore and centromeres, both in absence and presence of rapamycin (Fig. 5B, D). This approach enables us to gain valuable insights into the behaviour and dynamics of individual components.

8. FACS analysis of the DNA content is a standard to analyze the cell cycle defects of *T. gondii* mutants. The authors should investigate the mutant phenotype using this tool.

We thank the reviewer for this suggestion and conducted the suggested experiment. We have now incorporated the results in Figure 4D (and supplementary Fig. S4). The additional data obtained from the DNA content analysis supports our microscopy observations, revealing abnormal morphology of the parasites in the forward versus side scatter plot (supplementary Fig. S4). Moreover, the histogram demonstrated a shift towards the right in DNA content, indicating ongoing DNA replication but an inability to complete proper karyokinesis in the induced knockout parasites.

Reviewer 3 Advance Summary and Potential Significance to Field:
In this study, Wagner et al described three homologues of UNC family proteins in the apicomplexan

© 2023. Published by The Company of Biologists under the terms of the Creative Commons Attribution License (https://creativecommons.org/licenses/by/4.0/).

parasite T. gondii. The authors showed that the UNC-50 protein TgUNC1 localised to the Golgi and appeared to be not essential for the parasite and the SUN domain protein TgSLP2 showed a signal throughout the parasite and its biological roles have not been found. In constrast, the second SUN domain protein named TgSLP1 was expressed in a cell cycle-dependent manner and colocalised with the mitotic spindle and the centrocone. The conditional knockout of TgSLP1 leads to failure of nuclear division and loss of centrosome integrity. The work is interesting and the data are quite convincing. However, this work requires some modifications and additionnal experiments that will improve the quality and impact of the paper.

We thank the reviewer for his time to critically assess our study and the positive criticism that helped to improve our manuscript. Please find our detailed response below.

Reviewer 3 Comments for the Author:
Here are my major concerns :
1. It is not clear why the two first proteins TgUNC1 and TgSLP2 whose characterization add nothing to the functionally importance of TgSLP1 should be shown as main figures. No speficic and important functions are discovered for these two first proteins despite many efforts to characterize them by confocal microsocpy and gene knockout. Therefore, I will suggest to show the data of TgUNC1 and TgSLP2 described in figures 1, 2 and 3 (out of 7 main figures) as supplemenatry figures. The authors could quickly and briefly indicated in the text and also in the discussion section that they do not discover any important functions for TgUNC1 and TgSPL2, therefore they focus their attention and work more on TgSLP1 that is an essential protein for parasite's nuclear division.

We agree with the reviewer, and we have put the characterisation of TgUNC1 and TgSLP2 into the supplementary section of the manuscript. As our intention was to search for SUN domain proteins in *Toxoplasma*, we have retained the overview of the three SUN-like proteins that could be found in the genome of the parasite in Figure 1.

2. The link between TgSLP1 and VPS31 is not clearly explained or no solid experimental evidence has been provided to justify why VPS31 should be studied and presented herein. In addition, the authors do not mention this protein later on in the discussion paragraph.

We found VPS31 in a parallel study on the ESCRTIII complex in *Toxoplasma* and were surprised to find that there is almost perfect colocalisation of VPS31 and TgSLP1. However, during the revision of this manuscript, we continued our analysis of ESCRTIII functions, which will now be the topic of an independent future study.
Therefore, we removed the VPS31 data from this manuscript.

3. I am wondering why electron microscopy (EM) has not been done to see the ultrastructural morphology and modifications that occured in the knock out TgSLP1 mutant. Only confocal microscopy and antibodies have been used and EM will be more informative to see the ultractural sections of mutant, for examples, the parasite's nucleus leaving the cytoplasm and being located between the plasmalemma and inner membrane complex (IMC) as stated by the authors, and even new phenotypes.

We appreciate the reviewer's suggestion. Based on our experience with EM imaging, we have determined that it would not provide us additional information in this particular case. Instead, we have chosen to utilise STED super-resolution microscopy to examine TgSLP1 and markers associated with the centrosome, kinetochore and centromeres, both in the absence and presence of rapamycin (Fig. 5B, D). This approach enables us to gain valuable insights into the cellular structures and their dynamics.

4. I am quite surprised that the authors have only relied on « educational guess » by looking at putative homologues in genome database of *T. gondii* instead of using the epitope tagged TgSLP1 transgenic parasites to identify TgSLP1 partners by cross-linking experiments following by mass spectrometry analyses to discover potential KASH-like proteins that seems to be absolutely required for the proper functions of TgSLP1. These kind of experiments will seriously improve the impact of this work.

© 2023. Published by The Company of Biologists under the terms of the Creative Commons Attribution License (https://creativecommons.org/licenses/by/4.0/).

We made multiple attempts to identify interaction partners using TurboID and subsequent mass spectrometry, but unfortunately did not get any candidates in a reliable manner. We mention this now in the manuscript (lines 330-333). We decided to mention the accession number of some of our candidates as additional information for the reader, but (since these candidates were not linked to SLP1) decided not to present detailed data.

Minor concerns :

5. Fig S2 depicted diagrams of the strategies used to invalide these genes and I am not sure if this is neccessary to be shown.

As the main focus in this work is on TgSLP1, we fully agree with the reviewer and have removed supplementary Figure S2 from the manuscript as suggested.

6. Is there any guess that the classical ATc-inducible knock out system may work when LoxP, U1SnRNP- meduiated silencing and auxin-inducible degron system failed for TgSLP2 ?

We tried to insert a LoxP site upstream of the TgSLP2 locus to excise the gene using the DiCre-system. Despite several attempts at different positions in the gene, we could not include the LoxP which is only few base pairs long. Based in this experience, we believe that even a complete replacement of the promoter is not successful. Still, this is a good suggestion and worth trying. Currently, our guess is that genetic manipulations of this locus are difficult due to the presence of another (potentially) critical gene, TGGT1_207110 (phenotypic score of - 3.68) that is upstream of *slp2*. Consequently, promoter replacement would be as problematic as inserting a LoxP site into this locus.

7. The GRA1 confocal micrsocopy shown in Fig S1 in not convincing. Normally, the GRA1 signal usually decorates very nicely the PVM and it was not the case here.

We agree with the reviewer. It seems that we had problems with the antibody, so we have removed this image from the supplementary Figure S1.

Otherwise, my opinion is that this manuscript is interesting but requires a revision in its presentation and additional experiments before publication in JCS.

We thank the reviewers for their helpful comments and hope that the revised manuscript is now acceptable for publication in JCS.

Second Decision Letter

MS ID#: JOCES/2022/260337

MS TITLE: The SUN-like protein TgSLP1 is essential for nuclear division in the apicomplexan parasite Toxoplasma gondii

AUTHORS: Mirjam Wagner, Yuan Song, Elena Jimenez-Ruiz, Sonja Haertle, and Markus Meissner
ARTICLE TYPE: Research Article

I am happy to tell you that your manuscript has been accepted for publication in Journal of Cell Science, pending standard ethics checks.

© 2023. Published by The Company of Biologists under the terms of the Creative Commons Attribution License (https://creativecommons.org/licenses/by/4.0/).
